# Safety and efficacy of single-dose primaquine to interrupt *Plasmodium falciparum* malaria transmission in children compared with adults: a systematic review and individual patient data meta-analysis

**DOI:** 10.1016/S1473-3099(25)00078-7

**Published:** 2025-09

**Authors:** Daniel Yilma, Kasia Stepniewska, Teun Bousema, Chris Drakeley, Prashanti Eachempati, Philippe J Guerin, Andreas Mårtensson, Richard Mwaiswelo, Walter R Taylor, Karen I Barnes, WWARN Paediatric Primaquine for P falciparum Transmission Blocking Study Group, Elizabeth N Allen, Eliana Arango, Karen I. Barnes, Guido J.H. Bastiaens, Teun Bousema, Joelle Brown, Rutuja Chhajed, Robert J. Commons, Umberto D'Alessandro, Nicholas PJ Day, Mehul Dhorda, Alassane Dicko, Arjen Dondorp, Chris Drakeley, Lek Dysoley, Prashanti Eachempati, Badria El-Sayed, Salah-Eldin El-Zaki, Alice C Eziefula, Roly Gosling, Philippe J Guerin, Gordon Guyatt, Muzamil M. Abdel Hamid, Eli Harriss, Wasif Ali Khan, Titus K Kwambai, Benedikt Ley, Andreas Mårtensson, Lwidiko E. Mhamilawa, Dominic Mosha, Richard Mwaiswelo, Billy Ngasala, Roland Ngu, Joseph Okebe, Eugenie Poirot, Ric N Price, Sumayyah Rashan, Caitlin Richmond, Aaron M Samuels, Farhad Shokraneh, Frank Smithuis, Kasia Stepniewska, William Stone, Inge Sutanto, Walter R Taylor, Feiko ter Kuile, Kamala Thriemer, Kyaw Myo Tun, Alfred B Tiono, Rob W van der Pluijm, Amelie Vantaux, Nicholas J White, Daniel Yilma

**Affiliations:** a Jimma University Clinical Trial Unit, Department of Internal Medicine, Institute of Health, Jimma University, Jimma, Ethiopia; b Division of Clinical Pharmacology, Department of Medicine, University of Cape Town, Cape Town, South Africa; c WorldWide Antimalarial Resistance Network (WWARN) and Infectious Diseases Data Observatory (IDDO), University of Cape Town, Cape Town, South Africa; d WWARN and IDDO, Oxford, UK; e Department of Medical Microbiology, Radboud University Medical Center, Nijmegen, Netherlands; f Department of Infection Biology, London School of Hygiene & Tropical Medicine, London, UK; g MAGIC Evidence Ecosystem Foundation, Oslo, Norway; h Peninsula Dental School, University of Plymouth, Plymouth, UK; i Centre for Tropical Medicine and Global Health, Nuffield Department of Medicine, University of Oxford, Oxford, UK; j Global Health and Migration Unit, Department of Women's and Children's Health, Uppsala University, Uppsala, Sweden; k Department of Infectious Diseases, Uppsala University Hospital, Uppsala, Sweden; l Department of Microbiology, Immunology and Parasitology, School of Medicine, Kairuki University, Dar es Salaam, Tanzania; m Mahidol-Oxford Tropical Medicine Research Unit, Faculty of Tropical Medicine, Mahidol University, Bangkok, Thailand

## Abstract

**Background:**

Adding a single dose of primaquine to artemisinin-based combination therapy (ACT) for the treatment of falciparum malaria can reduce the transmission of *Plasmodium falciparum* and could limit the spread of artemisinin partial resistance, including in Africa, where the disease burden is greatest. We aimed to compare the safety and efficacy of single-dose primaquine plus ACT between young children (aged <5 years) and older children (aged 5 years to <15 years) and adults (aged ≥15 years), and between low and moderate-to-high transmission areas.

**Methods:**

For this systematic review and individual patient data meta-analysis, we searched PubMed, Embase, Web of Science, Cochrane Central Register of Controlled Trials, WHO Global Index Medicus, OpenGrey.eu, ClinicalTrials.gov, and the WHO International Clinical Trials Registry Platform, from database inception to April 3, 2024, with no language restrictions. We included prospective studies on efficacy against falciparum malaria that enrolled at least one child younger than 15 years and involved a study group given a single dose of primaquine (≤0·75 mg/kg) plus ACT. Studies involving mass drug administration, healthy volunteers, or patients with severe malaria or mixed (with non-falciparum) infections were excluded. For inclusion in the efficacy analysis, data on transmission potential (as determined by gametocytaemia, infectivity, or both) at enrolment and follow-up (day 3, day 7, or day 14) were required; the safety analysis required data on haemoglobin concentrations or haematocrit values at enrolment and at one or more follow-up visits by day 7, any data on adverse events, or both. After independent screening of the search results by two reviewers, the investigators of eligible studies were invited to contribute individual patient data. We quantified day 7 gametocyte carriage, probability of infecting a mosquito, decreases (>25%) in haemoglobin concentration associated with anaemia, and adverse events until day 28 using regression analyses, with random study-site intercepts to account for clustered data. These analyses were registered with PROSPERO, CRD42021279363 (safety) and CRD42021279369 (efficacy).

**Findings:**

Of 5697 records identified by the search, 30 studies were eligible for analysis. Of these, individual patient data were shared for 23 studies, including 6056 patients from 16 countries: 1171 (19·3%) young children (aged <5 years), 2827 (46·7%) older children (aged 5 years to <15 years), and 2058 (34·0%) adults (aged ≥15 years). Adding a single low dose of primaquine (0·2–0·25 mg/kg) to ACTs reduced day 7 gametocyte positivity (adjusted odds ratio [aOR] 0·34, 95% CI 0·22–0·52; p<0·001) and infectivity to mosquitoes over time (aOR per day 0·02, 0·01–0·07, p<0·001). No difference was found in the effect of single low-dose primaquine both on gametocyte positivity in young children compared with older children (1·08, 0·52–2·23; p=0·84) and adults (0·50, 0·20–1·25; p=0·14) and between low-transmission and moderate-to-high transmission settings (1·07, 0·46–2·52; p=0·86), and on infectivity to mosquitoes in young children compared with older children (1·36, 0·07–27·71; p=0·84) and adults (0·31, 0·01–8·84; p=0·50) and between low-transmission and moderate-to-high transmission settings (0·18, 0·01–2·95; p=0·23). Gametocyte clearance was also similar for different ACTs (dihydroartemisinin–piperaquine *vs* artemether–lumefantrine) when combined with a primaquine target dose of 0·25 mg/kg (1·56, 0·65–3·79; p=0·32 at day 7). However, patients given a primaquine dose of less than 0·2 mg/kg with dihydroartemisinin–piperaquine were more likely to have gametocytaemia than those treated with artemether–lumefantrine (5·68, 1·38–23·48; p=0·016 at day 7). There was no increase in anaemia-associated declines in haemoglobin concentration (>25%) at a primaquine dose of 0·25 mg/kg, regardless of age group, transmission setting, and glucose-6-phosphate dehydrogenase status. The risks of adverse events of grade 2 or higher and of serious adverse events were similar between primaquine and no-primaquine groups, including in young children.

**Interpretation:**

Regardless of malaria transmission intensity and age group, a single dose of 0·25 mg/kg primaquine is safe and efficacious for reducing *P falciparum* transmission. These findings underscore the need for primaquine formulations suitable for young children, and also provide supportive evidence to expand the use of single low-dose primaquine in regions with a moderate-to-high transmission rate that are threatened by artemisinin partial resistance.

**Funding:**

The EU and the Bill & Melinda Gates Foundation.

## Introduction

Primaquine is the only widely available antimalarial drug that kills mature *Plasmodium falciparum* gametocytes, the stage of the parasite lifecycle responsible for the transmission of malaria from humans to mosquitoes.^1^ Since 2012, WHO has recommended the addition of a single low dose of primaquine (0·25 mg/kg) to artemisinin-based combination therapy (ACT) in areas with artemisinin partial resistance (ART-R), to limit the spread of resistance strains, and in areas of low transmission, to reduce onward transmission of *P falciparum* malaria and to advance malaria elimination.^2^

Despite malaria-control efforts, including pivotal integrated vector control, young children (aged <5 years) remain highly susceptible to malaria infection and have the highest burden of clinical malaria.^3^ Because these infections are often associated with gametocytaemia, children contribute substantially to the infectious malaria reservoir.^4^, ^5^, ^6^ Furthermore, although the effectiveness of primaquine depends on achieving high coverage rates at a population level, no paediatric primaquine formulation has yet been marketed.^7^

**Research in context**

**Evidence before this study**

Artemisinin is the basis of current malaria treatments. However, artemisinin-resistant malaria is spreading—including in Africa, where most malaria cases and deaths occur—and young children are most affected. Primaquine is the only widely available antimalarial that kills mature *Plasmodium falciparum* gametocytes, the stage of the parasite lifecycle responsible for the transmission of malaria from humans to mosquitoes; however, no paediatric formulation of primaquine has yet been marketed. In our previous systematic review and individual patient data meta-analysis, we showed that the WHO-recommended single low dose of primaquine was safe and efficacious in all age groups. However, at that time, data were insufficient to enable a robust comparison between young children (aged <5 years) and older children (aged 5 years to <15 years) or adults (aged ≥15 years), or between areas of low and moderate-to-high malaria transmission. We searched PubMed, Embase, Web of Science, the Cochrane Central Register of Controlled Trials, WHO Global Index Medicus, OpenGrey.eu, ClinicalTrials.gov, and the WHO International Clinical Trials Registry Platform, from database inception to April 3, 2024, with no language restrictions; search terms included Medical Subject Headings and the free-text terms ((Primaquine OR Primacin) AND (Malaria OR Plasmodium)); for full details of each search, see the appendix (p 3). 5697 records were identified through this database search, from which 30 studies were eligible for analysis. Individual patient data were shared for 23 studies, involving 6056 patients from 16 countries. These patients included 1171 (19·3%) young children (aged <5 years); however, only 55 were infants (aged <1 year).

**Added value of this study**

This study pooled all available individual-patient data to provide a robust comparison of the safety and efficacy of a single low dose of primaquine in young children and in older children and adults, and between areas of low and moderate-to-high transmission. We found that, before treatment, gametocytes are more prevalent in young children than in older children and adults and in areas of moderate-to-high transmission than in areas of low transmission. Adding a single low dose of primaquine to artemisinin-based combination therapy for falciparum malaria is safe and efficacious, regardless of age and transmission intensity. We also showed that gametocyte clearance rates differ with different artemisinin-based combination therapies. When administered without primaquine or with a very low (<0·2 mg/kg) dose of primaquine, patients given dihydroartemisinin–piperaquine were more than five times more likely to have gametocytaemia than those treated with artemether–lumefantrine; however, gametocytaemia rates were similar when these therapies were combined with the WHO-recommended primaquine target dose of 0·25 mg/kg.

**Implications of all the available evidence**

Our analyses suggest that the WHO-recommended single low dose of primaquine (target dose 0·25 mg/kg) is as safe and efficacious in blocking the transmission of *P falciparum* malaria in young children as it is in older children and adults, in areas of both low and moderate-to-high transmission intensity. This finding has important implications for malaria treatment policies, providing supportive evidence to expand the use of single low-dose primaquine in areas threatened by artemisinin partial resistance—including moderate-to-high transmission areas in sub-Saharan Africa, where the effect of large-scale deployment of single low-dose primaquine on the transmission of artemisinin-resistant *P falciparum* parasites should be closely monitored and evaluated. The higher prevalence of gametocytaemia in children than in adults highlights the importance of marketing a user-friendly, affordable primaquine formulation suitable for young children, both in areas with drug-resistant malaria regardless of transmission intensity and in areas advancing towards malaria elimination.

At high total doses, primaquine can cause severe drug-induced haemolysis in individuals with glucose-6-phosphate dehydrogenase (G6PD) deficiency.^8^ However, WHO recommends that single low-dose primaquine can be given safely without G6PD testing. Previously, we reported the safety and efficacy of single low-dose primaquine in all age groups.^9^, ^10^ Young children seemed to be at the highest risk of anaemia, although insufficient data were available to enable a robust comparison between young children and older children (aged 5 years to <15 years) and adults (aged ≥15 years). Additional individual-participant data have since become available, allowing us to reassess the safety and efficacy of single primaquine doses up to 0·75 mg/kg for blocking *P falciparum* transmission—in young children compared with older children and adults and in areas of low transmission compared with areas of moderate-to-high transmission.

## Methods

### Search strategy and selection criteria

This systematic review and individual patient data meta-analysis included prospective clinical efficacy trials of patients with uncomplicated or asymptomatic *P falciparum* infection that enrolled at least one child younger than 15 years; had at least one study group receiving ACT plus a single primaquine dose of up to 0·75 mg/kg; and had data on patient demographics and primaquine use, timing, and dose. Mass drug administration studies and studies involving healthy volunteers or patients with severe malaria or mixed (with non-falciparum) infections were excluded.

For inclusion in the efficacy analysis, additional data were required on transmission potential (as determined by gametocytaemia, detected by molecular methods [quantitative nucleic acid-based amplification or quantitative RT-PCR]), infectivity (measured by membrane-feeding assays at enrolment and follow-up [at least one of day 3, day 7, and day 14]), or both. For inclusion in the safety analysis, we required data on haemoglobin concentrations or haematocrit values at enrolment and at least one other follow-up visit by day 7, data on adverse events, or both.

We searched PubMed, Embase, Web of Science, the Cochrane Central Register of Controlled Trials, WHO Global Index Medicus, OpenGrey.eu, ClinicalTrials.gov, and the WHO International Clinical Trials Registry Platform. The search was first conducted on Jan 3, 2020, and was updated on April 3, 2024, with a start date of database inception in each case and no restrictions on language. We used Medical Subject Headings and the free-text terms ((Primaquine OR Primacin) AND (Malaria OR Plasmodium)); for full details of each search, see the appendix (p 3).

After automated and manual removal of duplicate search results in EndNote X9 (Clarivate; Philadelphia, PA, USA), the titles and abstracts of retrieved studies were independently screened in Covidence (Veritas Health Innovation; Melbourne, VIC, Australia) by two of three reviewers (RB, CR, and SR). Two reviewers (EA and DY) then each independently screened the full text of selected studies. Any discrepancies regarding the eligibility of a study were resolved by a third reviewer (KIB). The investigators of eligible studies were then invited to contribute their individual patient data and participate in the WorldWide Antimalarial Resistance Network (WWARN) Paediatric Primaquine for *P falciparum* Transmission Blocking Study Group. De-identified data were collated in the WWARN secure repository using standardised methods.^11^ This study presents the combined results from two protocols, was conducted according to the PRISMA Individual Patient Data statement (appendix p 4), and is registered with PROSPERO, CRD42021279363 (safety)^12^ and CRD42021279369 (efficacy).^13^

### Data analyses

Efficacy analyses were conducted separately for patients with and without baseline gametocytaemia, as defined by the presence of gametocytes detected in the blood by molecular methods in each study. For gametocyte carriage on day 7 and day 14, logistic regression analyses were fitted with random intercepts for study site. Microscopy density measures were included for studies that used molecular methods to detect only gametocyte positivity. For samples positive by a molecular method but with zero microscopy count, density was assumed to be half the microscopy detection limit (8 gametocytes per μL). Data from membrane-feeding experiments were analysed using logistic regression to identify predictors of a patient infecting at least one mosquito. Random intercepts accounted for multiple measurements per patient or clustering within each membrane-feeding experiment. All models included age group (<5 years, 5 years to <15 years, and ≥15 years), primaquine dose (mg/kg), and log_10_ baseline gametocyte density as covariates. Other predefined covariates we adjusted for, if appropriate, were sex, baseline log_10_ asexual parasite density, haemoglobin concentration, nutritional status, G6PD status (classified as per primary study), ACT, gametocyte-measurement method, and malaria transmission intensity. G6PD status was mostly assessed by qualitative methods, using either a fluorescent spot test or Carestart rapid diagnostic tests. The transmission intensity of areas was defined on the basis of estimates of the *P falciparum* prevalence rate, with low transmission defined as study sites with a prevalence rate of less than 0·15 and moderate-to-high transmission as study sites with rates of 0·15 or higher. The probability of gametocyte carriage on day 7 and day 14 for patients receiving different primaquine doses with artemether–lumefantrine or dihydroartemisinin–piperaquine was estimated using a fractional polynomial model.

Linear regression models were fitted for the absolute reduction in haemoglobin concentrations between day 0 and day 7, and logistic regression models were fitted for risk of a decrease of more than 25% in haemoglobin concentration by day 7 with concomitant either moderate-to-severe anaemia (haemoglobin <10 g/dL) or severe anaemia (haemoglobin <7 g/dL). Random intercepts for study site were included to account for within-study clustering. Two-way and three-way interaction terms between G6PD status, age group, and primaquine dose were used to explore whether the dose effect differed between age groups and G6PD status. Primaquine dose was analysed either as a continuous variable or categorised on the basis of target dose (0·0625–0·125 mg/kg, 0·2–0·25 mg/kg, 0·4–0·5 mg/kg, or 0·75 mg/kg). The following predefined covariates were adjusted for, if appropriate: baseline haemoglobin concentration, asexual parasitaemia, gametocytaemia, nutritional status, sex, G6PD status, fever, ACT, and transmission intensity. Haematological recovery was assessed by comparing the mean change in haemoglobin concentrations between day 0 and days 21 or 28. All analyses were conducted with Stata version 17.0.

Adverse events were classified using the Medical Dictionary for Regulatory Activities and analysed by frequency, severity, and relatedness to primaquine. For each adverse event group, the proportions of patients affected by age group, and with and without primaquine, were calculated with exact binomial 95% CIs.

We used GRADE^14^ to define the certainty of evidence for predefined outcomes when comparing patients enrolled in randomised controlled trials (RCTs) who received ACT with primaquine (0·25 mg/kg, range 0·15–0·38) or without primaquine.

### Role of the funding source

The funders of the study had no role in study design, data collection, data analysis, data interpretation, or writing of the report.

## Results

**Figure 1:**
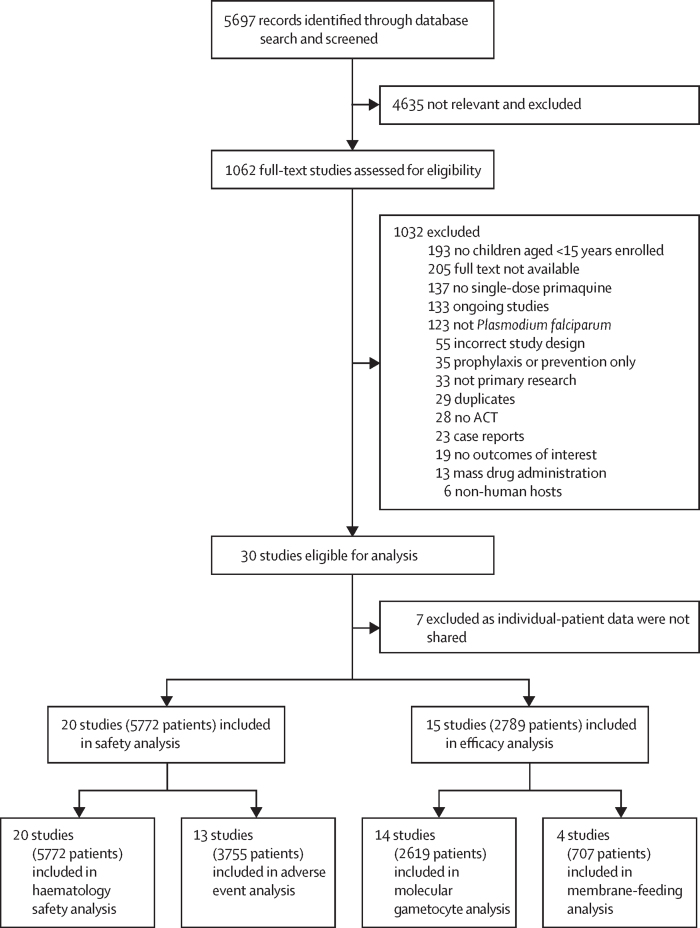
Study selection ACT=artemisinin-based combination therapy.

Of 1062 studies screened for full-text review, 30 were eligible for inclusion, of which individual patient data were available from 23 studies involving 6056 patients from 16 countries: 1171 (19·3%) were younger than 5 years, 2827 (46·7%) were aged between 5 years and <15 years, and 2058 (34·0%) were aged ≥15 years (figure 1). 19 studies were RCTs, two were open-label clinical trials, and two were prospective observational studies. 16 studies included a no-primaquine group and 16 studies were conducted in Africa. Apart from one study^15^ that used age-based primaquine dosing, all studies used primaquine target doses, which ranged from 0·0625 mg/kg to 0·75 mg/kg, although most studies included 0·25 mg/kg (14 studies) and 0·75 mg/kg (nine studies; appendix p 7).

14 studies from ten countries measured gametocytaemia by molecular methods, with data for 2449 (93·5%) of 2619 patients available for inclusion in the efficacy analysis (figure 1, appendix p 7). Seven studies used quantitative nucleic acid-based amplification and the remaining seven used quantitative RT-PCR, with target transcripts including Pfs25, Pfs230p, and Pfg377 mRNA.

At enrolment, gametocytes were detected in 1764 (72·0%) of 2449 patients. Baseline gametocytaemia was more prevalent in young children (421 [82·9%] of 508) than in older children (1039 [74·0%] of 1404; p<0·001) and adults (304 [56·6%] of 537; p<0·001), and was also more prevalent in moderate-to-high transmission settings (1009 [90·0%] of 1121) than in low-transmission settings (755 [56·9%] of 1328; p<0·001; appendix p 11).

Across all age groups and transmission settings, patients given primaquine were less likely to have gametocytes at day 7 and day 14 than patients given ACT alone (Table 1, Table 2; appendix pp 12–15). No difference was observed in the effect of single low-dose primaquine on day 7 gametocyte positivity in young children compared with older children (adjusted odds ratio [aOR] 1·08, 95% CI 0·52–2·23; p=0·84) and adults (0·50, 0·20–1·25; p=0·14), and between low-transmission and moderate-to-high transmission settings (1·07, 0·46–2·52; p=0·86; appendix p 16). At day 7 and day 14, patients with higher baseline gametocyte densities were more likely to have gametocytes, whereas patients with baseline hyperparasitaemia and higher haemoglobin concentrations were less likely to have gametocytes (table 1).

**Table 1:** Multivariable mixed-effects logistic regression for gametocyte positivity on day 7 and day 14 in patients with detectable gametocytaemia on day 0

		Day 7 gametocyte positivity*(N=1537, n=511, 11 studies)		Day 14 gametocyte positivity*(N=1255, n=265, nine studies)	
		aOR (95% CI)	p value	aOR (95% CI)	p value
Sex					
	Male	1·38 (1·07–1·79)	0·014	1·03 (0·72–1·46)	0·89
	Female	Ref	..	Ref	..
	Baseline log_10_ gametocytaemia*	1·95 (1·68–2·27)	<0·001	1·97 (1·63–2·38)	<0·001
Baseline parasitaemia					
	>10^5^ parasites per μL	0·24 (0·13–0·46)	<0·001	0·30 (0·11–0·82)	0·019
	≤10^5^ parasites per μL	Ref	..	Ref	..
	Baseline haemoglobin concentration, g/dL	0·85 (0·78–0·94)	<0·001	0·96 (0·85–1·09)	0·58
Age					
	<5 years	1·06 (0·61–1·84)	0·85	0·90 (0·42–1·95)	0·80
	5 to <15 years	0·75 (0·48–1·19)	0·23	0·82 (0·44– 1·52)	0·53
	≥15 years	Ref	..	Ref	..
Primaquine dose					
	None	Ref	..	Ref	..
	Very low (0·0625–0·125 mg/kg)	0·51 (0·27–0·97)	0·041	0·33 (0·12–0·89)	0·029
	Low (0·2–0·25 mg/kg)	0·34 (0·22–0·52)	<0·001	0·31 (0·14–0·66)	0·002
	Intermediate (0·4–0·5 mg/kg)	0·17 (0·09–0·29)	<0·001	0·27 (0·13–0·59)	0·001
	High (0·75 mg/kg)	0·24 (0·12–0·47)	<0·001	0·21 (0·07–0·67)	0·008
Artemisinin-based combination therapy					
	Artemether–lumefantrine	Ref	..	Ref	..
	Artesunate and sulfadoxine–pyrimethamine	2·42 (0·59–9·85)	0·22	5·70 (1·81–17·97)	0·003
	Dihydroartemisinin–piperaquine	3·87 (1·67–8·94)	0·002	8·48 (3·92–18·35)	<0·001
Transmission intensity†					
	Low	0·77 (0·36–1·66)	0·50	0·98 (0·46–2·08)	0·95
	Moderate to high	Ref	..	Ref	..

N is the number of patients included in the model and n is the number of patients with gametocyte positivity at the indicated timepoints. This model includes the interaction between primaquine dose and artemisinin-based combination therapy; see table 2. aOR=adjusted odds ratio.

* For studies in which only gametocyte positivity was determined by a molecular method, density measures by microscopy were included. For patients with positive samples by molecular methods and a zero microscopy count, density was assumed to be 8 gametocytes per μL (half of the detection limit by microscopy assuming microscopic quantification against 500 white blood cells or 1/16 μL).

† There was no significant difference in the effect of primaquine dose in different transmission settings in the same model (appendix pp 16–17).

**Table 2:** Gametocyte positivity on day 7 and day 14 in patients with detectable gametocytaemia on day 0 based on primaquine target dose and according to ACT

	**No primaquine**			**Very low-dose primaquine (0·0625–0·125 mg/kg)**			**Low-dose primaquine (0·2–0·25 mg/kg)**			**Intermediate-dose primaquine (0·4–0·5 mg/kg)**			**High-dose primaquine (0·75 mg/kg)**		
	n/N	aOR (95% CI)	p value	n/N	aOR (95% CI)	p value	n/N	aOR (95% CI)	p value	n/N	aOR (95% CI)	p value	n/N	aOR (95% CI)	p value
**Gametocyte positivity on day 7**															
Artemether–lumefantrine	135/300	Ref	..	24/92	Ref	..	56/198	Ref	..	24/173	Ref	..	15/138	Ref	..
Artesunate and sulfadoxine–pyrimethamine	31/42	2·42 (0·59–9·84)	0·22	..	..	..	..	−	..	..	..	..	8/46	0·77 (0·17–3·64)	0·75
Dihydroartemisinin–piperaquine	113/162	3·86 (1·67–8·93)	0·002	18/25	5·68 (1·38–23·48)	0·016	59/183	1·56 (0·65–3·79)	0·32	19/97	2·08 (0·74–5·86)	0·16	9/81	0·85 (0·25–2·86)	0·80
**Gametocyte positivity on day 14**															
Artemether–lumefantrine	40/204	Ref	..	6/90	Ref	..	11/141	Ref	..	11/124	Ref	..	4/81	Ref	..
Artesunate and sulfadoxine–pyrimethamine	27/41	5·70 (1·81–17·93)	<0·001	..	..	..	..	..	..	..	..	..	2/47	0·61 (0·09–4·43)	0·63
Dihydroartemisinin–piperaquine	101/154	8·47 (3·92–18·31)	<0·001	13/24	11·87 (2·72–51·89)	0·001	34/175	2·29 (0·88–5·96)	0·090	13/94	2·45 (0·79–7·55)	0·12	3/80	0·89 (0·16–5·08)	0·90

aOR estimates are also adjusted for sex, age, haemoglobin concentration, hyperparasitaemia and gametocytaemia at baseline, and transmission intensity. aOR=adjusted odds ratio.

**Figure 2:**
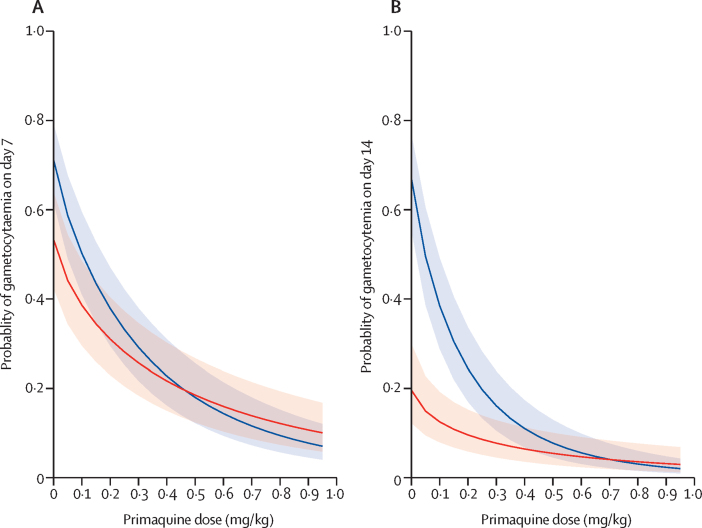
Predicted probability of gametocyte carriage 7 days and 14 days after treatment for different doses of primaquine Predicted probability of gametocytaemia on day 7 (A) and day 14 (B) after treatment initiation. The red lines represent individuals treated with artemether–lumefantrine and the blue lines represent individuals treated with dihydroartemisinin–piperaquine. Shaded areas correspond to 95% CIs. Median values for other variables were assumed in the models.

Notably, the rate of decline in gametocyte carriage associated with primaquine dose differed between patients treated with artemether–lumefantrine and those treated with dihydroartemisinin–piperaquine (figure 2). Of patients given less than 0·2 mg/kg primaquine, those treated with artemether–lumefantrine were less likely to have gametocytaemia at day 7 and day 14 than those treated with dihydroartemisinin–piperaquine. However, for patients who received a single dose of at least 0·25 mg/kg primaquine with artemether–lumefantrine or dihydroartemisinin–piperaquine, gametocyte carriage was similarly low on day 7 and day 14 (figure 2, table 2).

Adding single low-dose primaquine to ACT probably decreases malaria transmission, as inferred from gametocyte carriage by day 7 (table 3).

**Table 3:** Summary of findings

	**Study results and measurements (aOR, 95% CI)**	**Absolute effect estimates**			**GRADE certainty and quality of the evidence**	**Summary**
		ACT alone	ACT plus primaquine	Difference (95% CI)		
Outcome 1: malaria transmission as inferred from gametocyte carriage on day 7	0·23 (0·16–0·32); based on data from 725 participants in eight studies	514 per 1000	196 per 1000	318 fewer per 1000 (369 fewer to 261 fewer); magnitude of effect on malaria transmission is uncertain	Moderate, due to serious indirectness*	Primaquine (0·25 mg/kg) probably decreases malaria transmission in the first week after treatment
Outcome 2: malaria transmission as inferred from risk of patient infecting at least one mosquito	0·47 (0·24–0·92); based on data from 303 participants in three studies	267 per 1000	146 per 1000	121 fewer per 1000 (187 fewer to 16 fewer); magnitude of effect on malaria transmission is uncertain	Very low, due to very serious imprecision and very serious indirectness†	We are uncertain whether primaquine (0·25 mg/kg) decreases malaria transmission
Outcome 3: symptoms of anaemia as inferred from day 7 haemoglobin concentration <10 g/dL and >25% decrease in haemoglobin concentration	0·45 (0·19–1·02); based on data from 2303 participants in six studies	18 per 1000	8 per 1000	10 fewer per 1000 (15 fewer to 0 more)	Low, due to very serious imprecision‡	Primaquine (0·25 mg/kg) might not increase symptoms of anaemia
Outcome 4: adverse event of vomiting in first 3 days	0·79 (0·63–1·42); based on data from 1584 participants in eight studies	72 per 1000	58 per 1000	14 fewer per 1000 (25 fewer to 27 more)	Moderate, due to serious imprecision§	Primaquine (0·25 mg/kg) probably has little or no effect on vomiting in the first 3 days
Outcome 5: any other adverse events	0·96 (0·75–1·24); based on data from 1584 participants in eight studies	490 per 1000	480 per 1000	10 fewer per 1000 (71 fewer to 54 more)	Moderate, due to serious imprecision§	Primaquine (0·25 mg/kg) probably has little or no effect on other adverse events
Outcome 6: serious adverse events	0·79 (0·37–1·68); based on data from 863 participants in eight studies	36 per 1000	29 per 1000	7 fewer per 1000 (22 fewer to 23 more)	Moderate, due to serious imprecision§	Primaquine (0·25 mg/kg) probably has little or no effect on serious adverse events

The intervention ACT plus primaquine (0·25 mg/kg; range 0·15–0·38) was compared with ACT alone in patients with falciparum malaria. Outcomes were adjusted as follows: outcome 1 for sex, age, transmission intensity, schizontocidal treatment, baseline parasitaemia and gametocytaemia, and random effect of study site; outcome 2 for gametocytaemia, schizontocidal treatment, and transmission intensity; outcome 3 for age, glucose-6-phosphate dehydrogenase status, transmission intensity, baseline haemoglobin concentration, hyperparasitaemia, and random effect of study site; and outcomes 4, 5, and 6 for random effect of study site. ACT=artemisinin-based combination therapy. aOR=adjusted odds ratio.

* Indirectness: very serious. Rated down one level as gametocyte carriage is a surrogate outcome for indicating transmission of malaria (it is the only widely available measure for transmission currently).

† Imprecision: very serious**.** Rated down two levels as the sample of included studies does not satisfy optimal information size (sample size needed: 1688) and the ratio of the upper to the lower boundary of the CI is >2·5 (ratio=3·83); Indirectness: very serious. Rated down two levels as the risk of a patient infecting at least one mosquito is a surrogate outcome for indicating infectivity (thereby leading to more transmission).

‡ Imprecision: very serious. Rated down two levels as the sample of included studies does not satisfy optimal information size (sample size needed: 33 124) and the ratio of the upper to the lower boundary of the CI is >2·5 (ratio=5·37).

§ Imprecision: serious. Rated down one level as the width of the CI is consistent with both important benefit and harm (crossing 1). Although the initial assessment of risk of bias was high, the point estimate suggests fewer side-effects with the intervention and the bias would increase the estimate; therefore, we did not rate down for risk of bias.

Four studies reported mosquito-feeding experiments, three provided data on patient infectiousness (ie, infected at least one mosquito) and the proportion of mosquitoes infected between day 0 and day 14, and one study reported patient infectiousness at day 7. A single 0·25 mg/kg dose of primaquine was tested in all four studies, a no-primaquine group was included in three studies, and other primaquine doses were tested in two studies. Patients were treated with either artemether–lumefantrine (two studies) or dihydroartemisinin–piperaquine (two studies). Two studies included children younger than 15 years and two included patients older than 5 years. Two studies were conducted in low-transmission areas and two in moderate-to-high transmission areas.

Greater reduction in mosquito infectivity over time was observed with a primaquine target dose of 0·25 mg/kg than with doses of 0·0625–0·125 mg/kg; higher primaquine doses had similar effects to 0·25 mg/kg (appendix p 18). The risk of mosquitoes becoming infected with the use of single low-dose primaquine was not different in young children than in older children (aOR 1·36, 95% CI 0·07–27·71; p=0·84) and adults (0·31, 0·01–8·84; p=0·50), and between low-transmission and moderate-to-high transmission settings (0·18, 0·01–2·95; p=0·23; table 4). The risk of a patient infecting at least one mosquito and the percentage of mosquitoes infected with primaquine use were strongly associated with gametocyte density at the time of mosquito feeding (appendix p 18). Mosquito infectivity decreased over time with artemether–lumefantrine alone but not with dihydroartemisinin–piperaquine alone. However, with a primaquine dose of 0·25 mg/kg, the risk of infecting at least one mosquito was zero by day 3 regardless of the type of ACT (data not shown).

**Table 4:** The effect of primaquine on the probability of a patient infecting at least one mosquito and the probability of a mosquito being infected in membrane experiments in patients with gametocytaemia at baseline and at the time of sampling

		**No primaquine**			**Very low-dose primaquine (0·0625–0·125 mg/kg)**			**Low-dose primaquine (0·25 mg/kg)**			**Intermediate-dose primaquine (0·4–0·5 mg/kg)**		
		n/N	aOR (95% CI)	p value	n/N	aOR (95% CI)	p value	n/N	aOR (95% CI)	p value	n/N	aOR (95% CI)	p value
**Patient infecting at least one mosquito (N=251 patients, n=531 feeds, three studies)**													
Age													
	<5 years	4/29	0·13 (0·01–1·08)	0·059	..	..	..	2/14	0·81 (0·06–10·46)	0·87	1/15	0·34 (0·01–9·11)	0·52
	5 to <15 years	21/88	0·25 (0·05–1·16)	0·078	38/84	2·27 (0·50–10·39)	0·29	17/91	0·55 (0·11–2·69)	0·46	14/65	1·25 (0·21–7·09)	0·82
	≥15 years	18/44	Ref	..	8/37	Ref	..	11/37	Ref	..	6/27	Ref	..
Transmission intensity													
	Low	1/15	0·01 (0·0004–0·31)	0·008	..	..	..	2/26	0·18 (0·02–1·65)	0·13	..	..	..
	Moderate to high	42/146	Ref	..	46/121	..	..	28/116	Ref	..	21/107	..	..
**Mosquito becomes infected (N=30 535 mosquitoes, n=531 feeds, 251 patients, three studies)**													
Age													
	<5 years	44/1264	0·05 (0·003–0·71)	0·028	..	..	..	9/634	0·31 (0·01–8·84)	0·50	27/676	0·29 (0·004–19·65)	0·56
	5 to <15 years	104/4531	0·07 (0·01–0·47)	0·006	414/5937	1·41 (0·21–9·36)	0·72	278/5224	0·23 (0·03–1·61)	0·14	122/3494	1·03 (0·11–9·48)	0·98
	≥15 years	191/2593	Ref	..	101/2233	Ref	..	301/2204	Ref	..	56/1745	Ref	..
Transmission intensity													
	Low	0/730	..	..	..	..	..	52/1302	0·18 (0·01–2·95)	0·23	..	..	..
	Moderate to high	335/7658	Ref	..	515/8170	..	..	536/6760	Ref	..	205/5915	..	..

All estimates adjusted for artemisinin-based combination therapy and gametocytaemia at the time of sampling.

Whether adding single low-dose primaquine to ACT decreases malaria transmission is uncertain, as inferred from the risk of a patient infecting at least one mosquito (table 3).

The haematological safety analysis included data from 5772 patients in 20 studies spanning 14 countries (figure 1, appendix p 19). Of 1169 young children included, 55 (0·95%) were infants (aged 6 months to <1 year). G6PD status was assessed in 16 studies (appendix pp 7–8) with results known for 4243 (73·5%) of 5772 patients; 131 (12·1%) of 1085 young children, 202 (9·0%) of 2246 older children, and 50 (5·5%) of 912 adults were G6PD-deficient. All young children deficient in G6PD and all children (<15 years) with intermediate G6PD activity were from study sites in Africa.

A single primaquine dose was given to 4066 (70·4%) of 5772 patients. Except for one large study^15^ that used age-based dosing (median actual dose 0·21 mg/kg, range 0·07–0·41), all study groups used target weight-based doses ranging from 0·0625 mg/kg to 0·75 mg/kg. Patient characteristics at enrolment were generally similar between age groups and between primaquine and no-primaquine groups for each age category. However, young children were more commonly hyperparasitaemic and anaemic than were older children and adults and, in adult males, hyperparasitaemia was more common in the primaquine group than in the no-primaquine group (appendix p 19). We observed no difference in severe anaemia at day 7 between the primaquine and no-primaquine groups for each age category (appendix p 22).

An increase in primaquine dose of 0·1 mg/kg was not associated with a decrease in haemoglobin concentration of more than 25% alongside moderate-to-severe anaemia (haemoglobin <10 g/dL) by day 7 across age groups, transmission settings, and in patients with normal or unknown G6PD activity (table 5, appendix pp 24–26). Primaquine at a dose of 0·25 mg/kg might not increase symptoms of anaemia in the first week after starting treatment (table 3).

**Table 5:** Risk factors for change in haemoglobin concentration on day 7 after the first dose of artemisinin-based combination therapy

		Decrease in Hb concentration of >25% plus moderate-to-severe anaemia*in patients with baseline Hb >10 g/dL (N=3183, n=56)		Decrease in Hb concentration of >25% plus moderate-to-severe anaemia*in all patients (N=3865, n=63)	
		aOR (95% CI)	p value	aOR (95% CI)	p value
Age					
	<5 years†	7·93 (2·08–30·33)	0·002	5·75 (1·64–20·21)	0·006
	5 to <15 years	3·69 (1·17–11·68)	0·026	3·05 (1·05–8·85)	0·040
	Female ≥15 years	3·94 (1·17–13·20)	0·026	3·60 (1·18–11·00)	0·025
	Male ≥15 years	Ref	..	Ref	..
Increase in primaquine dose (0·1 mg/kg) by G6PD status					
	Normal	1·06 (0·96–1·81)	0·26	1·08 (0·97–1·19)	0·16
	Deficient	1·42 (0·97–2·08)	0·073	1·42 (0·99–2·04)	0·053
	Intermediate	..‡	..	..‡	..
	Unknown	1·07 (0·73–1·58)	0·73	1·19 (0·86–1·66)	0·30
Baseline haemoglobin concentration (g/dL)		1·23 (1·00–1·51)	0·050	1·24 (1·05–1·46)	0·009
Baseline parasitic load (counts per μL)					
	0§	Ref	..	Ref	..
	>0 to <100 000	1·71 (0·48–6·13)	0·41	1·66 (0·50–5·52)	0·41
	≥100 000	4·82 (1·13–20·49)	0·033	4·25 (1·08–16·71)	0·038
Transmission intensity					
	Low	2·35 (0·91–6·08)	0·077	2·48 (1·00–6·14)	0·049
	Moderate to high	Ref	..	Ref	..

N is the number of patients included in the sample and n is the number of patients with a decrease in Hb concentration. aOR=adjusted odds ratio. G6PD=glucose-6-phosphate dehydrogenase. Hb=haemoglobin.

* Moderate-to-severe anaemia is defined as Hb concentration <10 g/dL.

† A >25% decrease in haemoglobin concentration with anaemia by day 7 was reported in young children (15/524) and in male adults (5/537).

‡ No patient with intermediate G6PD activity had a decrease in Hb concentration of >25% plus moderate-to-severe anaemia.

§ Participants had gametocytes but no asexual forms at baseline.

However, the odds of a decrease in haemoglobin concentration of more than 25% by day 7 with moderate-to-severe anaemia were approximately eight times higher (aOR 7·93, 95% CI 2·08–30·33) in young children than in adult males (table 5). We found no association between primaquine dose and a decrease in haemoglobin concentration by day 7 in different transmission settings or across age groups, except in older children, in whom a 0·1 mg/kg increase in primaquine dose was associated with a very small (0·02 [95% CI 0·0001–0·04] g/dL) decrease in haemoglobin concentration (appendix p 26). For G6PD-deficient patients (n=341; four studies), each 0·1 mg/kg increase in primaquine dose was associated with a 0·18 (0·09–0·26) g/dL decrease in haemoglobin concentration by day 7 (appendix p 26). Severe anaemia by day 7 was associated with lower baseline haemoglobin concentration, hyperparasitaemia, and low transmission intensity but not with age group, G6PD status, or primaquine dose (appendix p 26).

Of 3750 patients with haemoglobin measurements on days 0, 2 or 3, and 7, 2159 (57·6%) had a nadir of haemoglobin concentration on day 2 or day 3, regardless of age group, primaquine use, or G6PD status. The mean haemoglobin concentration increased similarly in both primaquine and no-primaquine groups by day 21 or day 28, across all age groups, and regardless of G6PD status (appendix pp 27–28).

13 studies with 3755 patients were included in the adverse event analysis (figure 1). 1860 (49·5%) of 3755 patients had at least one adverse event and 64 (1·7%) patients had at least one serious adverse event within 28 days. Nine studies (including 2282 patients) were RCTs with a no-primaquine group and four studies (1473 patients) did not have a no-primaquine group (two studies with randomisation of ACT and two observational studies).

In the nine RCTs, 1020 (44·7%) of 2282 patients had at least one adverse event, with adverse events occurring in 326 (64·7%) of 504 young children, 554 (40·9%) of 1356 older children, and 140 (33·2%) of 422 adults (p<0·001). In all age groups, the risks of adverse events of grade 2 or higher, serious adverse events, and adverse events of special interest (vomiting and gastrointestinal disturbances) were generally similar in patients who received primaquine and in those who did not (appendix pp 29–30).

34 (1·5%) of 2282 patients reported 35 serious adverse events within 28 days in the RCTs with a no-primaquine group (appendix pp 31–34). Of these 35 events, eight were considered possibly related to primaquine (seven decreases in haemoglobin concentration and one report of vomiting), 12 were considered unrelated or unlikely to be related to primaquine, and six were from one study that did not report relatedness. 22 of the serious adverse events were haematological, and all 22 were reported in three studies and within 7 days of ACT initiation. These haematological serious adverse events were based on a protocol-defined criterion of a decrease in haemoglobin concentration of more than 25% in two studies, and on the definition of serious adverse events according to the International Council for Harmonisation of Technical Requirements for Pharmaceuticals for Human Use guidelines^16^ in one study. In one study, ten patients (of whom five received placebo and five received primaquine) had blood transfusions within the first week; two of these events were considered related to primaquine by the authors of the primary study. All serious adverse events were transient, and all patients recovered fully.

Little to no difference in the risk of vomiting, any other adverse event, and serious adverse events was found between the primaquine and no-primaquine groups (table 3).

In the four studies without no-primaquine groups, 727 (49·4%) of 1473 patients reported adverse events and 18 (1·6%) of 1111 patients reported serious adverse events within 7 days of ACT initiation, with no serious adverse events reported in young children. Overall, three deaths were reported; all were considered unrelated to primaquine by the authors of the primary study, with one considered possibly related to dihydroartemisinin–piperaquine. One patient had a pre-existing immune-compromised condition and died on admission day 2; one patient died 10 days after being incorrectly enrolled and treated with artemether–lumefantrine and single low-dose primaquine despite a negative malaria test; and one patient died after developing a rash and respiratory distress after the first dose of dihydroartemisinin–piperaquine.

Across all age groups, the risk of haemoglobinuria was similar for patients in the primaquine and no-primaquine groups, and was also independent of the dose of primaquine (appendix pp 35–37).

None of the included studies were primarily designed to compare the safety and efficacy of primaquine between young children, older children, and adults (appendix pp 38–39); of the eight studies that were eligible but not included in the analysis (either because individual patient data were not shared [n=7] or because the study was ongoing [n=1]), all reported adverse events but only one reported membrane-feeding experiments and haematology safety data (appendix p 40).

## Discussion

This systematic review and individual patient data meta-analysis, which included more than 6000 patients from 23 studies in 16 countries, provides robust support for the very good safety and efficacy of single low-dose primaquine for *P falciparum* transmission blocking, in all age groups and in areas of low and moderate-to-high transmission intensity.

At enrolment, gametocytaemia was more prevalent in moderate-to-high transmission areas than in low transmission areas, and more prevalent in young children than in older children or adults. The efficacy of single low-dose primaquine in reducing day 7 and day 14 gametocytaemia was similar in young children and in older children and adults. This finding highlights the urgent need for a paediatric primaquine formulation for *P falciparum* transmission blocking, particularly given the higher prevalence of gametocytes in young children and the risks associated with administering inappropriately high doses when giving adult formulations to young children.

Patients treated with dihydroartemisinin–piperaquine were eight times more likely to have gametocytaemia on day 14 than those given artemether–lumefantrine, when the ACT was administered either alone or with a very low dose of primaquine. However, the difference was not significant with a primaquine dose greater than 0·2 mg/kg. A significant reduction in mosquito infectivity was observed with artemether–lumefantrine but not with dihydroartemisinin–piperaquine. Previous studies^17^, ^18^, ^19^ also showed that artemether–lumefantrine has a better gametocyte clearing effect than dihydroartemisinin–piperaquine, but less than that of artesunate–mefloquine. Although the long half-life of dihydroartemisinin–piperaquine is considered advantageous, adding single low-dose primaquine should improve its effectiveness in advancing malaria elimination efforts and limiting the spread of resistant parasites.

ART-R has emerged and is now spreading in several African countries with moderate-to-high transmission.^17^, ^18^, ^20^ ART-R results in delayed parasite clearance, with potentially increased prevalence and density of gametocytes even without subsequent recrudescence.^1^ Such effects would facilitate greater transmission of resistant than sensitive parasites,^19^ as seen previously with other antimalarials.^21^ Artemisinin drug pressure exacerbates the spread of partially resistant parasites, as more sensitive than resistant parasites are killed in treated infections.^19^ This individual patient data meta-analysis shows that, among patients seeking treatment, baseline gametocytaemia was much more prevalent in moderate-to-high transmission settings (90·0%) than in low-transmission settings (56·9%; p<0·001). Our findings support that the potent efficacy of primaquine is consistent regardless of malaria transmission intensity. As such, even in moderate-to-high transmission areas threatened by ART-R, adding single low-dose primaquine has the potential to reduce the further selection of resistant parasites by artemisinin-based treatments and counteract the greater transmissibility of artemisinin-resistant parasites.^22^ This finding is consistent with the WHO 2012 recommendation to add a single low dose of primaquine (0·25 mg/kg) to ACT in areas threatened by ART-R and in malaria elimination areas.^2^

WHO revised this guidance in 2015^23^ to recommend single low-dose primaquine only in low-transmission areas; this revision was based on the concern that reducing malaria transmission requires a high coverage of primaquine, which is hindered by a large reservoir of asymptomatic parasite carriers in areas of moderate-to-high transmission. However, control of asymptomatic infections in areas threatened by ART-R is best achieved by strengthening integrated vector control and preventive treatment strategies that are not artemisinin-based, such as vaccines and seasonal malaria chemoprevention, to decrease the prevalence of asymptomatic malaria without selecting for artemisinin-resistant parasites.^24^

Malaria frequently results in a decrease in haemoglobin concentration through the destruction of red blood cells (both those containing and not containing parasites) and bone marrow dyserythropoiesis.^25^, ^26^ Young children are more prone to develop anaemia after malaria infection, anaemia-associated nutritional deficiencies, and intestinal helminthic infections than are older children and adults.^27^, ^28^ Drugs that can result in iatrogenic haemolysis could increase the severity of anaemia. However, our analysis showed that a single primaquine dose did not result in changes in haemoglobin concentration in patients with normal or intermediate G6PD activity, with similar effects seen in young children as in older children or adults. In patients with G6PD deficiency, all of whom were from Africa, each 0·1 mg/kg increase in primaquine dose was associated with a small (0·18 g/dL) decrease in haemoglobin concentration and a 33% increase in moderate-to-severe anaemia, which is reassuringly transient. These data support the safety of the WHO-recommended single target dose of primaquine (0·25 mg/kg, range 0·15–0·38 mg/kg). Although young children had more adverse events and serious adverse events than older children and adults, there was no association with primaquine use in controlled studies. Similarly, no differences in vomiting, any gastrointestinal disturbance, and haemoglobinuria were observed with primaquine use in any age category.

Although, to our knowledge, this is the largest analysis to date on the safety and efficacy of single low-dose primaquine for *P falciparum* malaria transmission blocking, our study has several limitations. No data were available on the safety and tolerability of repeated single low-dose primaquine use for recurrent *P falciparum* infection, which remains a knowledge gap that is particularly relevant to areas of high transmission intensity. Nevertheless, the findings from vivax malaria research on repeated primaquine dosing offer reassuring evidence.^29^ Gametocytes are the sexual stages of the malaria parasites capable of infecting mosquitoes, and are the most widely used predictor of *P falciparum* transmission blocking efficacy. However, assessing gametocyte carriage—even when using sensitive molecular methods—underestimates the sterilising effects of primaquine, as many infections with low gametocyte density are not transmitted.^30^ Transmission-blocking effects are therefore often found to be greater in membrane-feeding experiments and infectivity studies than in gametocyte clearance studies, yet the relatively small number of such infectivity studies published testifies to their difficulty, particularly in young children. Only two of the four studies on membrane feeding included young children; more data in individuals from this age group would therefore strengthen the evidence of efficacy in this key population. Even in this large individual patient data meta-analysis, we had little data on infants (aged 6 months to <1 year; n=55). Few (n=8) young children deficient in G6PD received primaquine doses greater than 0·25 mg/kg, so we were not able to estimate the effect of higher primaquine doses on haemoglobin concentration in this vulnerable population.

This systematic review and individual patient data meta-analysis corroborated that adding the WHO-recommended target dose of 0·25 mg/kg primaquine to ACTs is safe and effective as a *P falciparum* malaria transmission-blocking tool, in all age groups and in areas of low and moderate-to-high transmission intensity. When given without primaquine or with a very low dose of primaquine, artemether–lumefantrine has better gametocytocide and transmission-blocking effects than dihydroartemisinin–piperaquine. The higher prevalence of gametocytaemia in young children than in adults highlights the importance of developing a user-friendly, affordable paediatric primaquine formulation for young children for the reduction of *P falciparum* transmission in areas threatened by ART-R, including sub-Saharan Africa, and in pre-elimination areas.

### WWARN Paediatric Primaquine for *P falciparum* Transmission Blocking Study Group

### Contributors

### Data sharing

Pseudonymised participant data used in this analysis are available for access via the WWARN website (https://www.iddo.org/wwarn/accessing-data). Requests for access will be reviewed by a data access committee to ensure that use of data protects the interests of the participants and researchers according to the terms of ethics approval and principles of equitable data sharing. Requests can be submitted by email to malariaDAC@iddo.org via the data access form. WWARN is registered with the Registry of Research Data Repositories.

## Declaration of interests

We declare no competing interests.
