# Supplementary material for: Safety and efficacy of single-dose primaquine to interrupt Plasmodium falciparum malaria transmission in children compared with adults: a systematic review and individual patient data meta-analysis
Source: Lancet Infect Dis. 2025 Sep;25(9):965–76. doi: 10.1016/S1473-3099(25)00078-7 (PMC12353880; doi:10.1016/S1473-3099(25)00078-7)
Supplement: Supplementary appendix [file mmc1.pdf]

THE LANCET
Infectious Diseases

Supplementary appendix

This appendix formed part of the original submission and has been peer reviewed. We post it as supplied by the authors.

Supplement to: Yilma D, Stepniewska K, Bousema T, et al. Safety and efficacy of single-dose primaquine to interrupt *Plasmodium falciparum* malaria transmission in children compared with adults: a systematic review and individual patient data meta-analysis. *Lancet Infect Dis* 2025; published online April 23. https://doi.org/10.1016/S1473-3099(25)00078-7.

APPENDIX

Yilma D, Stepniewska K, et al, Safety and efficacy of single-dose primaquine to interrupt *Plasmodium falciparum* malaria transmission in children compared with adults: a systematic review and individual patient data meta-analysis

Page

Text S1. Supplementary methods ... 3

Checklist S1. PRISMA-IPD Checklist of items to include when reporting a systematic review and meta-analysis of individual participant data (IPD) ... 4

Table S1. Studies included in analysis ... 7

Table S2. Baseline characteristics by age groups for efficacy study ... 9

Table S3: Baseline prevalence of gametocytes (detected by molecular methods), by age group and malaria transmission intensity ... 11

Figure S1. Forest plots of difference in proportions of participants with gametocytes (risk difference) on each day of follow-up. ... 12

Table S4. Gametocyte positivity using molecular methods (QT-NASBA or RT-PCR), by follow-up day and transmission intensity. ... 15

Table S5. Effect of primaquine dose on gametocyte positivity on Days 7 (A) and 14 (B) among patients with detectable gametocytemia on Day 0 across age group and transmission setting ... 16

Table S6. Mixed effects logistic regression for probability of a patient infecting at least 1 mosquito and probability of a mosquito being infected in membrane experiments conducted on blood taken within 14 days from treatment in patients with gametocytaemia at baseline and at the time of sampling ... 18

Table S7. Baseline characteristics of patients included in haematology safety analysis. ... 19

Table S8. Patients with >25% fractional decrease in haemoglobin and/or anaemia at day 3 and day 7 in patients with haemoglobin > 10 g/dl at baseline. ... 21

Table S9. Patients with >25% fractional decrease in haemoglobin or anaemia at day 3 and day 7 (including patients with baseline anaemia) ... 22

Figure S2. Proportion of patients at day 7 (A) with >25% fractional decrease in haemoglobin (B) Moderate-to-severe anemia (Hb < 10g/dL) by G6PD status, and age/sex category ... 23

Table S10. Risk factors for change in haemoglobin concentration on day 7 after first dose of ACT administration ... 25

Table S11. The effect of primaquine dose on haemoglobin change across age group and transmission settings ... 26

Figure S3. Mean with 95% Confidence interval haemoglobin 21 or 28 days after initiation of ACT ... 27

Table S12: Summary of adverse events by time since primaquine dosing and age categories in 9 controlled studies with a no-primaquine arm ... 29

Table S13: Serious adverse events reported within 28 days of ACT with or without primaquine administration. ... 31

Text S2. Supplementary results 35
Figure S4. Forest plot for hemoglobinuria by age group 36
Table S14. Factors associated with hemoglobinuria with logistic regression 37
Table S15. Risk of bias assessment in randomised controlled studies 38
Table S16. Risk of bias assessment in single arm observational studies 39
Table S17. Eligible studies not included 40
References 41

Text S1. Supplementary methods

Search term

The search terms employed were ‘primaquine’, ‘primacin’, ‘malaria’ OR ‘plasmodium’. Search strings using these terms were adapted to the database searched, with the following string used in Pubmed:

> (((((((Primaquine[Title/Abstract] OR Primacin[Title/Abstract]) OR ("Primaquine"[Mesh])))) OR primaquine[Title/Abstract])) AND ((((malaria[Title/Abstract] OR plasmodium[Title/Abstract]) OR (("Malaria"[Mesh]) OR "Plasmodium"[Mesh])))))

Definitions

Gametocytes on enrollment is be defined as any sexual parasitaemia count/presence within 24hrs of the reading, in patients in whom this was assessed by QT-NASBA or RT-PCR.
Gametocyte carriage during follow-up is defined as patIent gametocytaemia after enrollment (>24hrs) up to day 14 of follow-up, whilst taking account of reinfection rates, transmission levels, and concurrent asexual parasitaemia results within patients.
The appearance of gametocytes will be defined as gametocyte carriage during study follow-up in patients with no detectable gametocytes present at enrollment (within first 24hrs).
Prevalence of gametocytes during follow-up will be determined on days 3, 7, 14 according to patient gametocytaemia by QT-NASBA or RT-PCR on each day of observation. Patients with missing counts on that day will be excluded from the analysis, unless a missing count is between two positive counts (it will be assumed to be positive).
Infectiousness prevalence is defined as proportion of individuals infecting at least one mosquito.
Absolute reduction in Hb between times t_1 and t_2: as $hb(t_2)- hb(t_1)$
Fractional reduction in Hb between times t_1 and t_2: $(hb(t_2)- hb(t_1))/hb(t_1)$, or where $hb(t_i)$ denotes measured or estimated Hb at time t_i
Anaemia:

- Moderate (Hb ≥7 g/dL and <10g/dl)
- Severe (Hb <7g/dL)

Adverse Events (AEs): Any unfavourable medical occurrence in a trial participant. The adverse event does not necessarily have a causal relationship with the treatment, according to the ICH E6 guidelines. Grading classification and causality assessment of adverse events were as assessed by the primary study, and standardised as mild (grade 1), moderate (grade 2), severe (grade 3) and life-threatening (grade 4).

Serious adverse events (SAEs): any untoward medical occurrence that at any dose: results in death; is life-threatening; requires inpatient hospitalisation or results in prolongation of existing hospitalisation; results in persistent or significant disability/incapacity; is a congenital anomaly/birth defect; or is a medically important event or reaction. We will also include SAEs defined according to individual study-based reports

Transmission Intensity areas defined based on estimates of P. falciparum prevalence rate (PfPR)[1], assuming low transmission for study sites with a PfPR <0.15, moderate transmission if PfPR 0.15 to <0.40 and high transmission if PfPR ≥0.40
G6PD Status will be classified as severely deficient (<30% activity or a positive qualitative test (eg FST)) vs normal (>=30% activity) or a negative qualitative test (eg FST)). A second categorisation will be explored to assess patients with intermediate deficiency: severely deficient (<30% activity or a positive qualitative test (eg FST)), intermediate deficiency (>=30% to <70% activity) or normal (>=70% activity).
The nutritional status of children aged <5 years of age will be calculated as a weight-for-age z-score, using the igrowup package developed by WHO. Those with weight-for-age z-scores < -2 (i.e. below the 3rd centile) will be classified as underweight-for-age (termed underweight).
Data integrity, study group governance and ethics
The Study Group comprised a coordinating team and principal investigators (and/or their designees) who contributed relevant data sets of which they retained ownership[2]. Data were obtained in accordance with laws and ethical approvals applicable to the countries where studies were conducted and were deidentified before or during curation within the WWARN repository. The Oxford University Research Ethics Committee does not require review of the use of existing data that are anonymised and that cannot be traced back to individuals.
Risk of bias was assessed for included studies using the Cochrane Risk of Bias 2 tool[3] for randomised controlled trials and the Joanna Briggs Institute Case Series tool[4] for single-arm studies.

Checklist S1. PRISMA-IPD Checklist of items to include when reporting a systematic review and meta-analysis of individual participant data (IPD)

PRISMA-IPD Section/topic	Item No	Checklist item	Reported on page
**Title**			
Title	1	Identify the report as a systematic review and meta-analysis of individual participant data.	1
**Abstract**			
Structured summary	2	Provide a structured summary including as applicable:	See Summary
		**Background**: state research question and main objectives, with information on participants, interventions, comparators and outcomes.	
		**Methods**: report eligibility criteria; data sources including dates of last bibliographic search or elicitation, noting that IPD were sought; methods of assessing risk of bias.	
		**Results**: provide number and type of studies and participants identified and number (%) obtained; summary effect estimates for main outcomes (benefits and harms) with confidence intervals and measures of statistical heterogeneity. Describe the direction and size of summary effects in terms meaningful to those who would put findings into practice.	
		**Discussion:** state main strengths and limitations of the evidence, general interpretation of the results and any important implications.	
		**Other:** report primary funding source, registration number and registry name for the systematic review and IPD meta-analysis.	
**Introduction**			
Rationale	3	Describe the rationale for the review in the context of what is already known.	See Introduction
Objectives	4	Provide an explicit statement of the questions being addressed with reference, as applicable, to participants, interventions, comparisons, outcomes and study design (PICOS). Include any hypotheses that relate to particular types of participant-level subgroups.	See Introduction
**Methods**			
Protocol and registration	5	Indicate if a protocol exists and where it can be accessed. If available, provide registration information including registration number and registry name. Provide publication details, if applicable.	See Methods - Search strategy and selection criteria
Eligibility criteria	6	Specify inclusion and exclusion criteria including those relating to participants, interventions, comparisons, outcomes, study design and characteristics (e.g. years when conducted, required minimum follow-up). Note whether these were applied at the study or individual level i.e. whether eligible participants were included (and ineligible participants excluded) from a study that included a wider population than specified by the review inclusion criteria. The rationale for criteria should be stated.	See Methods - Search strategy and selection criteria
Identifying studies - information sources	7	Describe all methods of identifying published and unpublished studies including, as applicable: which bibliographic databases were searched with dates of coverage; details of any hand searching including of conference proceedings; use of study registers and agency or company databases; contact with the original research team and experts in the field; open adverts and surveys. Give the date of last search or elicitation.	See Methods - Search strategy and selection criteria
Identifying studies - search	8	Present the full electronic search strategy for at least one database, including any limits used, such that it could be repeated.	Text S1
Study selection processes	9	State the process for determining which studies were eligible for inclusion.	See Methods - Search strategy and selection criteria

Data collection processes	10	Describe how IPD were requested, collected and managed, including any processes for querying and confirming data with investigators. If IPD were not sought from any eligible study, the reason for this should be stated (for each such study).<br><br>If applicable, describe how any studies for which IPD were not available were dealt with. This should include whether, how and what aggregate data were sought or extracted from study reports and publications (such as extracting data independently in duplicate) and any processes for obtaining and confirming these data with investigators.	See Methods - Search strategy and selection criteria
Data items	11	Describe how the information and variables to be collected were chosen. List and define all study level and participant level data that were sought, including baseline and follow-up information. If applicable, describe methods of standardising or translating variables within the IPD datasets to ensure common scales or measurements across studies.	See Methods - Search strategy and selection criteria
IPD integrity	A1	Describe what aspects of IPD were subject to data checking (such as sequence generation, data consistency and completeness, baseline imbalance) and how this was done.	Text S1
Risk of bias assessment in individual studies.	12	Describe methods used to assess risk of bias in the individual studies and whether this was applied separately for each outcome. If applicable, describe how findings of IPD checking were used to inform the assessment. Report if and how risk of bias assessment was used in any data synthesis.	Table S1
Specification of outcomes and effect measures	13	State all treatment comparisons of interests. State all outcomes addressed and define them in detail. State whether they were pre-specified for the review and, if applicable, whether they were primary/main or secondary/additional outcomes. Give the principal measures of effect (such as risk ratio, hazard ratio, difference in means) used for each outcome.	See Methods – Data analyses
Synthesis methods	14	Describe the meta-analysis methods used to synthesise IPD. Specify any statistical methods and models used. Issues should include (but are not restricted to):<br>• Use of a one-stage or two-stage approach.<br>• How effect estimates were generated separately within each study and combined across studies (where applicable).<br>• Specification of one-stage models (where applicable) including how clustering of patients within studies was accounted for.<br>• Use of fixed or random effects models and any other model assumptions, such as proportional hazards.<br>• How (summary) survival curves were generated (where applicable).<br>• Methods for quantifying statistical heterogeneity (such as I^2 and t^2).<br>• How studies providing IPD and not providing IPD were analysed together (where applicable).<br>• How missing data within the IPD were dealt with (where applicable).	See Methods – Data analyses
Exploration of variation in effects	A2	If applicable, describe any methods used to explore variation in effects by study or participant level characteristics (such as estimation of interactions between effect and covariates). State all participant-level characteristics that were analysed as potential effect modifiers, and whether these were pre-specified.	See Methods – Data analyses
Risk of bias across studies	15	Specify any assessment of risk of bias relating to the accumulated body of evidence, including any pertaining to not obtaining IPD for particular studies, outcomes or other variables.	Text S1
Additional analyses	16	Describe methods of any additional analyses, including sensitivity analyses. State which of these were pre-specified.	
**Results**			
Study selection and IPD obtained	17	Give numbers of studies screened, assessed for eligibility, and included in the systematic review with reasons for exclusions at each stage. Indicate the number of studies and participants for which IPD were sought and for which IPD were obtained. For those studies where IPD were not available, give the numbers of studies and participants for which aggregate data were available. Report reasons for non-availability of IPD. Include a flow diagram.	Figure 1
Study characteristics	18	For each study, present information on key study and participant characteristics (such as description of interventions, numbers of participants, demographic data, unavailability of outcomes, funding source, and if applicable duration of follow-up). Provide (main) citations for each study. Where applicable, also report similar study characteristics for any studies not providing IPD.	Table S1

IPD integrity	A3	Report any important issues identified in checking IPD or state that there were none.	See Results
Risk of bias within studies	19	Present data on risk of bias assessments. If applicable, describe whether data checking led to the up-weighting or down-weighting of these assessments. Consider how any potential bias impacts on the robustness of meta-analysis conclusions.	Table S15 & S16
Results of individual studies	20	For each comparison and for each main outcome (benefit or harm), for each individual study report the number of eligible participants for which data were obtained and show simple summary data for each intervention group (including, where applicable, the number of events), effect estimates and confidence intervals. These may be tabulated or included on a forest plot.	See Results
Results of syntheses	21	Present summary effects for each meta-analysis undertaken, including confidence intervals and measures of statistical heterogeneity. State whether the analysis was pre-specified, and report the numbers of studies and participants and, where applicable, the number of events on which it is based.	See Results
		When exploring variation in effects due to patient or study characteristics, present summary interaction estimates for each characteristic examined, including confidence intervals and measures of statistical heterogeneity. State whether the analysis was pre-specified. State whether any interaction is consistent across trials.	
		Provide a description of the direction and size of effect in terms meaningful to those who would put findings into practice.	
Risk of bias across studies	22	Present results of any assessment of risk of bias relating to the accumulated body of evidence, including any pertaining to the availability and representativeness of available studies, outcomes or other variables.	See Results
Additional analyses	23	Give results of any additional analyses (e.g. sensitivity analyses). If applicable, this should also include any analyses that incorporate aggregate data for studies that do not have IPD. If applicable, summarise the main meta-analysis results following the inclusion or exclusion of studies for which IPD were not available.	-
**Discussion**			
Summary of evidence	24	Summarise the main findings, including the strength of evidence for each main outcome.	See Discussion
Strengths and limitations	25	Discuss any important strengths and limitations of the evidence including the benefits of access to IPD and any limitations arising from IPD that were not available.	See Discussion
Conclusions	26	Provide a general interpretation of the findings in the context of other evidence.	See Discussion
Implications	A4	Consider relevance to key groups (such as policy makers, service providers and service users). Consider implications for future research.	See Discussion
**Funding**			
Funding	27	Describe sources of funding and other support (such as supply of IPD), and the role in the systematic review of those providing such support.	See Summary and Methods

© Reproduced with permission of the PRISMA IPD Group, which encourages sharing and reuse for non-commercial purpose

Table S1. Studies included in analysis

Study ID	PMID	Design	Location	Year	Arms	FU days	N	Age	G6PD Testing methods	Target dose	Included in IPD meta-analysis				
											Gam	Membrane feeding	AE	Hb	Haemglobinuria
1	29996844	RCT	Myanmar	2013-2015	3d AL, 3d AL/fish oil, 5d AL, 5d/fish oil (PQ all arms)	42	149	6 months to 65 years	Not applicable	0.25	No	No	Yes	No	No
2	27825738 & 25887344	RCT	The Gambia	2013-2015	DP +/- PQ 0.2, 0.4, 0.75	42	694	> 1 year	FST (Dimopolous)	0.2 0.4 0.75	Yes	No	Yes	Yes	Yes
3	Unpublished	Open label CT	Tanzania	2019-2020	AL + 0.25 PQ (single arm)	28	157	1 to 10 years	Not applicable	0.25	No	Yes	No	Yes	No
4	27565897 & 27287612	RCT	Tanzania	2014	AL +/- PQ 0.25	28	220	≥ 1 year	CareStart RDT (AccessBio)	0.25	Yes	No	Yes	Yes	Yes
5	28749756	RCT	Colombia	2010-2014	AL +/- PQ 0.75-d3 (eligible), 0.25-d1,2,3 (ineligible), 0.50-d1 + 0.25-d3 (ineligible)	7	19	4 to 77 years	Not applicable	0.75	Yes	No	No	No	No
6	29548285	RCT	Sudan	2015	AL +/- PQ 0.25	42	231	≥ 1 year	CareStart RDT (AccessBio)	0.25	No	No	No	Yes	No
7	28289025 & 26952094	RCT	Burkina Faso	2013-2014	AL +/- PQ 0.25 0.40	14	360	2 to 15 years	BinaxNOW RDT (Alere Inc.)	0.25 0.4	Yes	Yes	Yes	Yes	Yes
8	18074034	RCT	Sudan	2004	ASSP +/- PQ 0.75	14	86	≥ 6 months	Not applicable	0.75	Yes	No	No	Yes	No
9	Unpublished	RCT	Kenya	2014-2015	DP +/- PQ 0.125 0.25 0.40 0.75	42	54	1 ≤ 12 years	FST (Trinity, Biotech)	0.125, 0.25, 0.4, 0.75	Yes	No	Yes	Yes	Yes
10	32171078 & 31345710	RCT	Thailand, Cambodia, Vietnam, Myanmar, Laos, Bangladesh, India, DRC	2015-2018	DP, DPMQ, ASMQ, AL, ALAQ (PQ all arms)	42	1101	2 to 65 years	Not applicable	0.25	No	No	Yes	Yes	No
11	27197604 & 24239324 & 24913169	RCT	Uganda	2011	AL +/- PQ 0.1 0.4 0.75	28	454	1 to 10 years	FST (R&D Diagnostics)	0.1 0.4 0.75	Yes	No	No	Yes	Yes
12	29324864	RCT	The Gambia	2015-2016	DP, DP + PQ 0.25 (G6PD def/norm), DP PQ 0.4	28	61	≥ 10 years	CareStart RDT (AccessBio) and FST	0.25, 0.4	Yes	No	Yes	Yes	No

13	36462528	RCT	DRC, Uganda	2017-2020	AL or DP +/- PQ 6m−<1y 1·25 mg, 1−5y 2·5 mg, 6−9y 5 mg, 10−14y 7·5, and ≥ 15y 15 mg	42	1137	6 months to 11 years	PCR & RDT (not specified)	Age-based dosing	No	No	Yes	Yes	Yes
14	27450652	Cohort	Eswatini	2014-2015	AL + PQ 0.25 (single arm)	7	94	≥ 1 year	CareStart RDT (AccessBio)	0.25	No	No	Yes	Yes	No
15	17925871	RCT	Tanzania	2006	ASSP +/- PQ 0.75	42	102	3 to 15 years	PCR	0.75	Yes	No	No	Yes	No
16	23175563	RCT	Indonesia	2008-2010	DP +/- PQ 0.75	42	373	≥ 5 years	FST (Trinity, Biotech)	0.75	No	No	No	Yes	No
17	27036739	Open label CT	Myanmar	2013-2014	DP + PQ 0.25 (Single arm)	42	114	6 months to 65 years	Not applicable	0.25	No	No	No	Yes	No
18	31234865	RCT	South Africa	2016-2018	AL-PQ 0.25/AL	42	140	> 1 year	CareStart RDT (AccessBio)	0.25	Yes	No	Yes	Yes	No
19	28931236	RCT	Kenya	2014-2015	DP +/- PQ 0.25	14	114	5 to 15 years	Not applicable	0.25	Yes	No	No	Yes	No
20	31964380	RCT	Tanzania	2013-2015	AL +/- PQ 0.75 d0 or d2	14	107	3 to 17 years	CareStart RDT (AccessBio)	0.75	Yes	No	Yes	Yes	No
21	30871496 & 32179526	RCT	Cambodia	2015-2016	DP +/- PQ 0.25	28	109	≥ 1 year	CareStart RDT (AccessBio) and FST	0.25	Yes	Yes	No	Yes	No
22	27128675	Cohort	Bangladesh	2014-2015	AL + PQ 0.75	28	115	≥ 1 year	FST (Randox, UK)	0.75	No	No	Yes	No	No
23	26906747	RCT	Mali	2013-2014	DP+/-PQ 0.0625, 0.125, 0.25, 0.5	28	81	5 to 50 years	Colorimetric quantification (R&D Diagnostics)	0.0625, 0.125, 0.25, 0.5	Yes	Yes	Yes	Yes	Yes

RCT –Randomized control trials; CT-Clinical trials; ASSP – artesunate plus sulfadoxine-pyrimethamine; AL– artemether-lumefantrine; DP – dihydroartemisinin-piperaquine; PQ – primaquine, Hb- Haemoglobin, AE- Adverse events, FU- Follow up, DRC-Democratic Republic of the Congo, DPMQ-dihydroartemisinin-piperaquine-mefloquine, ASMQ- artesunate–mefloquine, ALAQ-artemether–lumefantrine- amodiaquine; RDT- Rapid diagnostic test; FST-fluorescence spot test

Table S2. Baseline characteristics by age groups for efficacy study

	<5 years				5-<15 years				>=15 years			
	No Primaquine		Primaquine		No Primaquine		Primaquine		No Primaquine		Primaquine	
**Baseline Characteristics**	**N**	**Median (Range) or n (%)**	**N**	**Median (Range) or n (%)**	**N**	**Median (Range) or n (%)**	**N**	**Median (Range) or n (%)**	**N**	**Median (Range) or n (%)**	**N**	**Median (Range) or n (%)**
Age, Years	167	3 [1 - 4.9]	359	3 [0 - 4.9]	515	9 [5 - 14]	1003	8 [5 - 14]	250	28 [15 - 84]	325	25 [15 - 84]
Sex, Male	167	76 [46]	357	195 [55]	515	305 [59]	1003	543 [54]	250	166 [66]	325	205 [63]
Underweight, WAZ <-2	157	21 [13]	329	39 [12]	8	0 [0]	22	4 [18]				
WAZ	157	-.6 [-3.8 - 14.1]	329	-.7 [-3.6 - 2.6]	8	-.1 [-1.5 - 1]	22	-.5 [-2.6 - 2]				
Temperature, ºC	143	37.3 [36 - 40.7]	289	37.2 [36 - 40.7]	412	36.7 [34.3 - 40.5]	888	36.7 [34.2 - 41]	184	37.2 [36 - 41]	271	36.7 [35.5 - 39.9]
Fever, To > 37.5oC	143	62 [43]	289	110 [38]	412	89 [22]	888	161 [18]	184	83 [45]	271	80 [30]
Haemoglobin (HB), g/dl	162	10.3 [6.8 - 13.5]	354	10.5 [7.6 - 15]	507	11.7 [7.6 - 16.4]	996	11.7 [6 - 17.7]	244	13.3 [8.7 - 19.7]	307	12.8 [8.1 - 18.7]
Anaemia HB < 10 g/dl	162	65 [40]	354	127 [36]	507	63 [12]	996	129 [13]	244	13 [5]	307	13 [4]
Parasitaemia, /µL	158	11480 [0 - 432000]	343	9820 [0 - 518180]	461	551 [0 - 281680]	951	382 [0 - 420000]	163	1706.5 [0 - 386800]	253	54 [0 - 308333.5]
Hyperparasitaemia, >105 /µL	158	20 [13]	343	53 [15]	461	11 [2]	951	48 [5]	163	7 [4]	253	2 [1]
G6PD-deficient	104	10 [10]	194	7 [4]	375	15 [4]	734	17 [2]	220	17 [8]	276	32 [12]
Presence of gametocytes												
Microscopy	50	7 [14]	36	6 [16.7]	154	70 [45.5]	230	102 [44.4]	144	19 [13.2]	183	22 [12]
QT-NASBA	103	88 [85.4]	188	157 [83.5]	338	270 [79.8]	685	510 [74.5]	75	49 [65.3]	139	96 [69.1]
RT-PCR	73	63 [86]	183	151 [82.5]	191	141 [73.8]	402	327 [81.3]	142	69 [48.6]	185	94 [50.8]
Schizontocidal treatment												
AL	167	115 [69]	359	245 [68]	515	217 [42]	1003	494 [49]	250	108 [43]	325	119 [37]
ASSP	167	29 [17]	359	25 [7]	515	53 [10]	1003	54 [5]	250	23 [9]	325	27 [8]
DP	167	23 [14]	359	89 [25]	515	245 [48]	1003	455 [45]	250	119 [48]	325	179 [55]
Primaquine target dose (mg/kg)												
0.1			359	54 [15.0]			1003	61 [6.1]			325	0 [0]

0.125			359	6 [1.7]			1003	2 [0.2]			325	0 [0]
0.2			359	22 [6.1]			1003	120 [12.0]			325	30 [9.2]
0.25			359	51 [14.2]			1003	255 [25.4]			325	159 [48.9]
0.4			359	99 [27.6]			1003	284 [28.3]			325	54 [16.6]
0.75			359	127 [35.4]			1003	281 [28.0]			325	82 [25.2]
Transmission Intensity												
Low	167	43 [26]	359	78 [22]	515	218 [42]	1003	478 [48]	250	209 [84]	325	318 [98]
Moderate	167	104 [62]	359	239 [67]	515	198 [38]	1003	409 [41]	250	3 [1]	325	2 [1]
High	167	20 [12]	359	42 [12]	515	99 [19]	1003	116 [12]	250	38 [15]	325	5 [2]

Table S3: Baseline prevalence of gametocytes (detected by molecular methods), by age group and malaria transmission intensity

	Age							
	< 5 years		5-<15 years		>15 years		All age	
	**N**	**n (%)**	**N**	**n (%)**	**N**	**n (%)**	**N**	**n (%)**
Low malaria transmission	134	89 (66.42)	697	402 (57.68)	497	264 (53.12)	1328	755 (56.85)
Moderate to high malaria transmission	374	332 (88.77)	707	637 (90.10)	40	40 (100)	1121	1009 (90.01)

Figure S1. Forest plots of difference in proportions of participants with gametocytes (risk difference) on each day of follow-up. (Only individuals with gametocytes at enrolment were included. Abbreviations: CI, confidence interval; PQ, primaquine; RD, risk difference.)

1a. Day 3 1b. Day 7 and 1c. Day 14 with each sub grouped for age < 5 years, 5-<15 years and >= 15 years

1a. Day 3

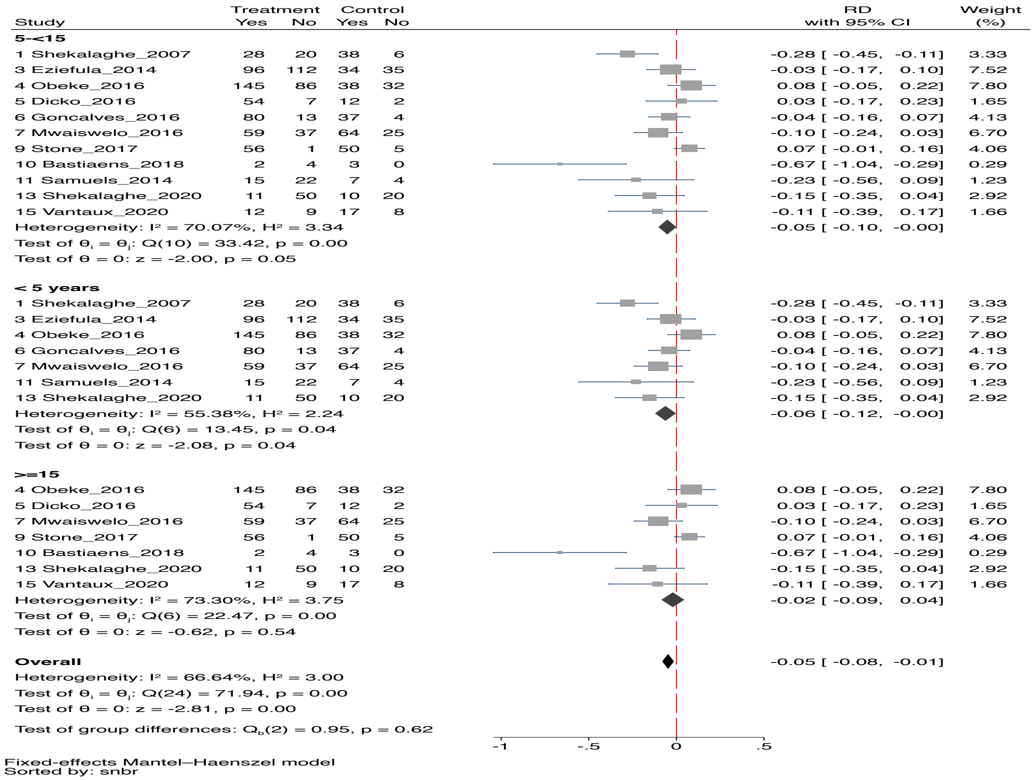

1b.Day 7

Study	Treatment Yes	Treatment No	Control Yes	Control No	RD with 95% CI	Weight (%)
**5-<15**						
1 Shekalaghe_2007	8	39	33	11	-0.58 [-0.75, -0.41]	2.73
2 El Sayed_2007	2	4	1	5	0.17 [-0.31, 0.65]	0.36
3 Eziefula_2014	43	204	36	53	-0.23 [-0.34, -0.12]	7.86
4 Obeke_2016	46	185	40	29	-0.38 [-0.51, -0.25]	6.39
5 Dicko_2016	35	25	14	0	-0.42 [-0.54, -0.29]	1.36
6 Goncalves_2016	39	160	49	46	-0.32 [-0.43, -0.21]	7.73
7 Mwaiswelo_2016	34	62	49	39	-0.20 [-0.34, -0.06]	5.52
9 Stone_2017	23	34	48	7	-0.47 [-0.62, -0.31]	3.36
10 Bastiaens_2018	0	5	2	0	-1.00 [-1.00, -1.00]	0.17
11 Samuels_2014	4	36	5	7	-0.32 [-0.61, -0.02]	1.11
12 Raman_2019	0	42	0	33	0.00 [0.00, 0.00]	2.22
13 Shekalaghe_2020	3	58	3	28	-0.05 [-0.16, 0.07]	2.47
15 Vantaux_2020	5	16	17	7	-0.47 [-0.73, -0.21]	1.35
Heterogeneity: I^2 = 74.34%, H^2 = 3.90					-0.30 [-0.34, -0.25]	
Test of $\theta_i = \theta_j$: Q(12) = 46.76, p = 0.00						
Test of θ = 0: z = -13.05, p = 0.00						
**< 5 years**						
1 Shekalaghe_2007	8	39	33	11	-0.58 [-0.75, -0.41]	2.73
2 El Sayed_2007	2	4	1	5	0.17 [-0.31, 0.65]	0.36
3 Eziefula_2014	43	204	36	53	-0.23 [-0.34, -0.12]	7.86
4 Obeke_2016	46	185	40	29	-0.38 [-0.51, -0.25]	6.39
6 Goncalves_2016	39	160	49	46	-0.32 [-0.43, -0.21]	7.73
7 Mwaiswelo_2016	34	62	49	39	-0.20 [-0.34, -0.06]	5.52
11 Samuels_2014	4	36	5	7	-0.32 [-0.61, -0.02]	1.11
13 Shekalaghe_2020	3	58	3	28	-0.05 [-0.16, 0.07]	2.47
Heterogeneity: I^2 = 80.56%, H^2 = 5.14					-0.29 [-0.34, -0.23]	
Test of $\theta_i = \theta_j$: Q(7) = 36.01, p = 0.00						
Test of θ = 0: z = -10.66, p = 0.00						
**>=15**						
2 El Sayed_2007	2	4	1	5	0.17 [-0.31, 0.65]	0.36
4 Obeke_2016	46	185	40	29	-0.38 [-0.51, -0.25]	6.39
5 Dicko_2016	35	25	14	0	-0.42 [-0.54, -0.29]	1.36
7 Mwaiswelo_2016	34	62	49	39	-0.20 [-0.34, -0.06]	5.52
9 Stone_2017	23	34	48	7	-0.47 [-0.62, -0.31]	3.36
10 Bastiaens_2018	0	5	2	0	-1.00 [-1.00, -1.00]	0.17
12 Raman_2019	0	42	0	33	0.00 [0.00, 0.00]	2.22
13 Shekalaghe_2020	3	58	3	28	-0.05 [-0.16, 0.07]	2.47
15 Vantaux_2020	5	16	17	7	-0.47 [-0.73, -0.21]	1.35
Heterogeneity: I^2 = 76.69%, H^2 = 4.29					-0.28 [-0.34, -0.22]	
Test of $\theta_i = \theta_j$: Q(8) = 34.32, p = 0.00						
Test of θ = 0: z = -9.57, p = 0.00						
**Overall**					-0.29 [-0.32, -0.26]	
Heterogeneity: I^2 = 75.35%, H^2 = 4.06						
Test of $\theta_i = \theta_j$: Q(29) = 117.66, p = 0.00						
Test of θ = 0: z = -19.32, p = 0.00						
Test of group differences: $Q_b(2)$ = 0.24, p = 0.89						

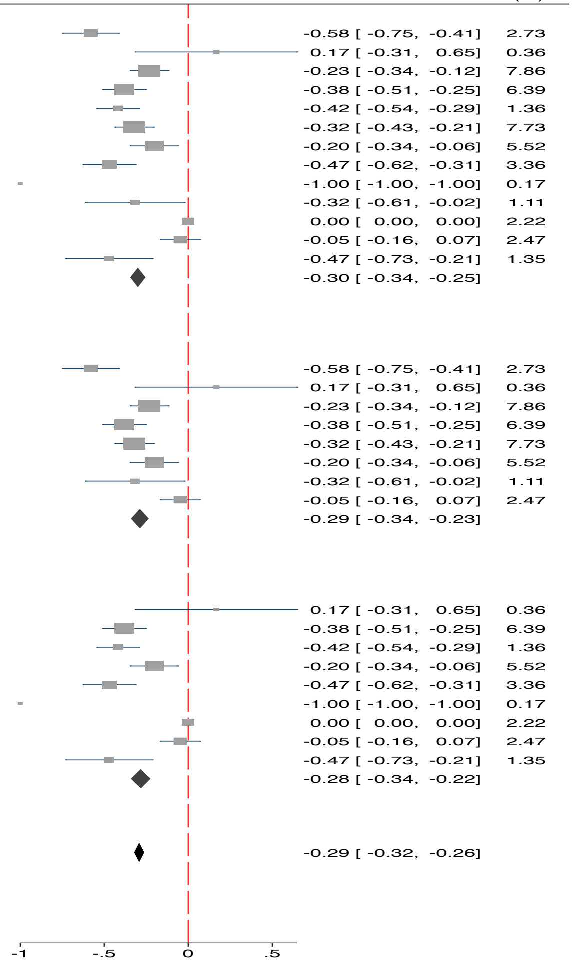

Fixed-effects Mantel–Haenszel model
Sorted by: snbr

1c. Day 14

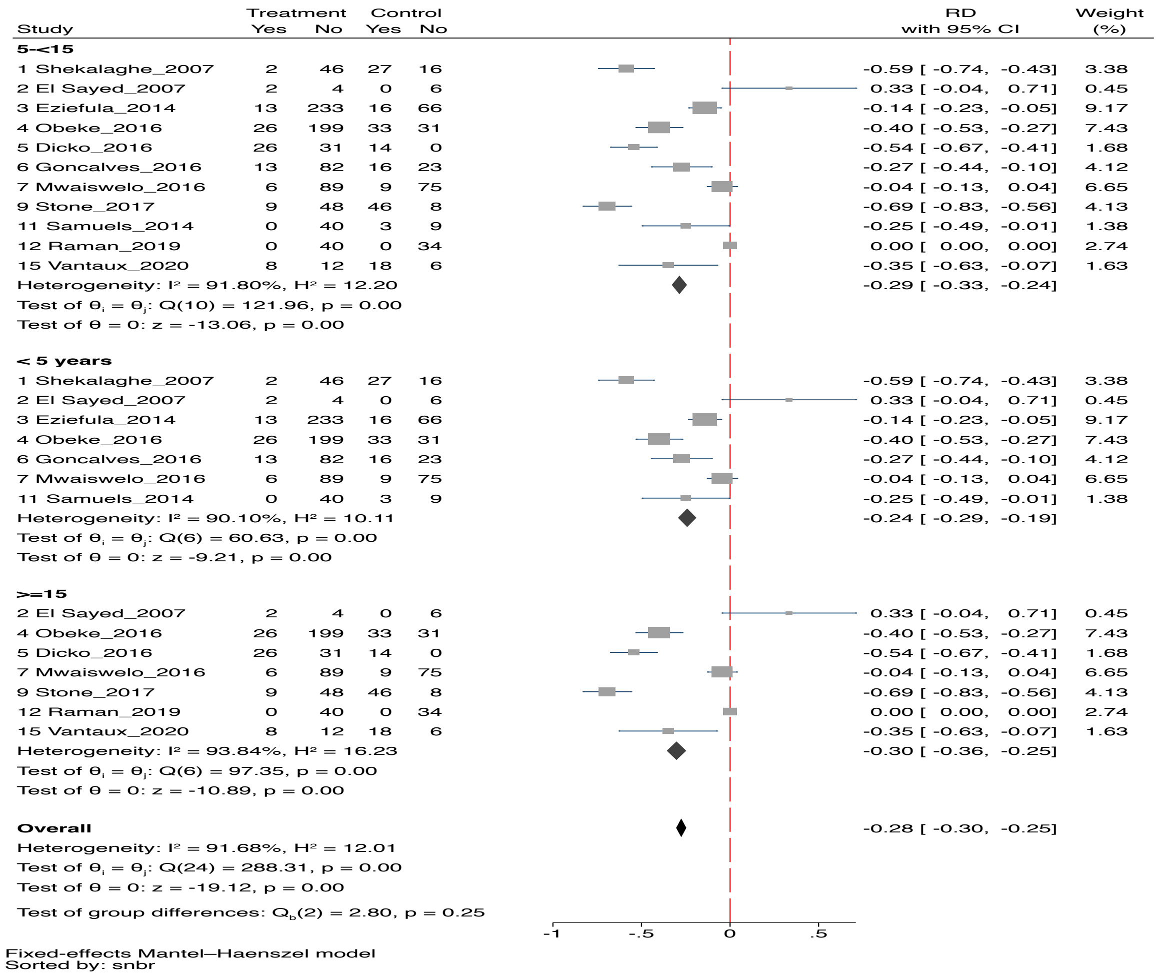
Treatment Control
Study Yes No Yes No
RD with 95% CI
Weight (%)
5-<15
1 Shekalaghe_2007 2 46 27 16 -0.59 [-0.74, -0.43] 3.38
2 El Sayed_2007 2 4 0 6 0.33 [-0.04, 0.71] 0.45
3 Eziefula_2014 13 233 16 66 -0.14 [-0.23, -0.05] 9.17
4 Obeke_2016 26 199 33 31 -0.40 [-0.53, -0.27] 7.43
5 Dicko_2016 26 31 14 0 -0.54 [-0.67, -0.41] 1.68
6 Goncalves_2016 13 82 16 23 -0.27 [-0.44, -0.10] 4.12
7 Mwaiswelo_2016 6 89 9 75 -0.04 [-0.13, 0.04] 6.65
9 Stone_2017 9 48 46 8 -0.69 [-0.83, -0.56] 4.13
11 Samuels_2014 0 40 3 9 -0.25 [-0.49, -0.01] 1.38
12 Raman_2019 0 40 0 34 0.00 [0.00, 0.00] 2.74
15 Vantaux_2020 8 12 18 6 -0.35 [-0.63, -0.07] 1.63
Heterogeneity: I² = 91.80%, H² = 12.20 -0.29 [-0.33, -0.24]
Test of θi = θj: Q(10) = 121.96, p = 0.00
Test of θ = 0: z = -13.06, p = 0.00
< 5 years
1 Shekalaghe_2007 2 46 27 16 -0.59 [-0.74, -0.43] 3.38
2 El Sayed_2007 2 4 0 6 0.33 [-0.04, 0.71] 0.45
3 Eziefula_2014 13 233 16 66 -0.14 [-0.23, -0.05] 9.17
4 Obeke_2016 26 199 33 31 -0.40 [-0.53, -0.27] 7.43
6 Goncalves_2016 13 82 16 23 -0.27 [-0.44, -0.10] 4.12
7 Mwaiswelo_2016 6 89 9 75 -0.04 [-0.13, 0.04] 6.65
11 Samuels_2014 0 40 3 9 -0.25 [-0.49, -0.01] 1.38
Heterogeneity: I² = 90.10%, H² = 10.11 -0.24 [-0.29, -0.19]
Test of θi = θj: Q(6) = 60.63, p = 0.00
Test of θ = 0: z = -9.21, p = 0.00
>=15
2 El Sayed_2007 2 4 0 6 0.33 [-0.04, 0.71] 0.45
4 Obeke_2016 26 199 33 31 -0.40 [-0.53, -0.27] 7.43
5 Dicko_2016 26 31 14 0 -0.54 [-0.67, -0.41] 1.68
7 Mwaiswelo_2016 6 89 9 75 -0.04 [-0.13, 0.04] 6.65
9 Stone_2017 9 48 46 8 -0.69 [-0.83, -0.56] 4.13
12 Raman_2019 0 40 0 34 0.00 [0.00, 0.00] 2.74
15 Vantaux_2020 8 12 18 6 -0.35 [-0.63, -0.07] 1.63
Heterogeneity: I² = 93.84%, H² = 16.23 -0.30 [-0.36, -0.25]
Test of θi = θj: Q(6) = 97.35, p = 0.00
Test of θ = 0: z = -10.89, p = 0.00
Overall -0.28 [-0.30, -0.25]
Heterogeneity: I² = 91.68%, H² = 12.01
Test of θi = θj: Q(24) = 288.31, p = 0.00
Test of θ = 0: z = -19.12, p = 0.00
Test of group differences: Qb(2) = 2.80, p = 0.25
-1 -.5 0 .5
Fixed-effects Mantel–Haenszel model
Sorted by: snbr

Table S4. Gametocyte positivity using molecular methods (QT-NASBA or RT-PCR), by follow-up day and transmission intensity.

Participants with gametocytes at enrolment												
	Primaquine						No primaquine					
	Transmission intensity						Transmission intensity					
	Low		Moderate		High		Low		Moderate		High	
	N	n(%)	N	n(%)	N	n(%)	N	n(%)	N	n(%)	N	n(%)
Day 0	490	490 (100.0)	291	291 (100.0)	400	400 (100.0)	265	265 (100.0)	175	175 (100.0)	143	143 (100.0)
Day 3[a]	425	234 (55.1)	216	164 (75.9)	288	165 (57.3)	250	131 (52.4)	145	127 (87.6)	89	51 (57.3)
Day 7	463	91 (19.7)	280	55 (19.6)	370	97 (26.2)	251	112 (44.6)	169	111 (65.7)	140	74 (52.9)
Day 14	386	42 (10.9)	222	22 (9.9)	321	21 (12.8)	212	60 (28.3)	143	91 (63.6)	101	31 (30.7)
**Participants with no detectable gametocytes at enrolment**												
	Primaquine						No primaquine					
	Transmission intensity						Transmission intensity					
	Low		Moderate		High		Low		Moderate		High	
	N	n(%)	N	n(%)	N	n(%)	N	n(%)	N	n(%)	N	n(%)
Day 0	366	0 (0.0)	9	0 (0.0)	70	0 (0.0)	207	0 (0.0)	10	0 (0.0)	23	0 (0.0)
Day 3[a]	289	57 (19.7)	7	0 (0.0)	22	7 (31.8)	159	40 (25.2)	9	3 (33.3)	14	1 (7.1)
Day 7	331	28 (8.5)	9	0 (0.0)	67	8(11.9)	193	30 (15.5)	10	3 (30.0)	22	3 (13.6)
Day 14	284	15 (5.3)	6	0 (0.0))	51	1 (1.9)	182	27 (14.8)	7	3 (42.8)	18	0 (0)

Table S5. Effect of primaquine dose on gametocyte positivity on Days 7 (A) and 14 (B) among patients with detectable gametocytemia on Day 0 across age group and transmission setting

A. Gametocyte Positivity on Days 7															
	No PQ			Very low dose PQ (0.0625-0.125 mg/kg)			low dose PQ (0.2-0.25 mg/kg)			Intermediate dose PQ (0.4-0.5 mg/kg)			High dose PQ (0.75 mg/kg)		
	**n/N**	**AOR (95% CI)**	**P-value**	**n/N**	**AOR (95% CI)**	**P-value**	**n/N**	**AOR (95% CI)**	**P-value**	**n/N**	**AOR (95% CI)**	**P-value**	**n/N**	**AOR (95% CI)**	**P-value**
**Age, years**#															
< 5	80/132	1.72 (0.80, 3.68)	0.16	14/49	2.94 (0.29, 30.23)	0.36	14/54	0.50 (0.20, 1.25)	0.14	12/73	1.12 (0.28, 4.43)	0.87	13/85	4.75 (0.53, 42.51)	0.16
5-<15	166/309	0.92 (0.48, 1.74)	0.79	27/67	3.89 (0.46, 32.23)	0.21	77/266	0.47 (0.24, 0.92)	0.03	27/175	0.89 (0.25, 3.18)	0.87	18/160	3.89 (0.46, 33.23)	0.21
>15	33/63	Ref		1/1			24/61			4/22			1/20		
Transmission setting*															
Low transmission	108/210	0.63 (0.28, 1.40)	0.25		-	-	59/195	1.07 (0.46, 2.52)	0.86	17/81	0.85 (0.30, 2.40)	0.76	12/135	0.40 (0.12, 1.23)	0.11
Moderate to high transmission	171/294	Ref		42/117	Ref		56/186	Ref		26/189	Ref		20/130	Ref	

**B. Gametocyte Positivity on Days 14**															
	**No PQ**			**Very low dose PQ (0.0625-0.125 mg/kg)**			**low dose PQ (0.2-0.25 mg/kg)**			**Intermediate dose PQ (0.4-0.5 mg/kg)**			**High dose PQ (0.75 mg/kg)**		
	**n/N**	**AOR (95% CI)**	**P-value**	**n/N**	**AOR (95% CI)**	**P-value**	**n/N**	**AOR (95% CI)**	**P-value**	**n/N**	**AOR (95% CI)**	**P-value**	**n/N**	**AOR (95% CI)**	**P-value**
Age, years#															
< 5	34/107	0.87 (0.34, 2.26)	0.78	4/46	0.75 (0.05, 11.01)	0.84	5/43	0.86 (0.22, 3.39)	0.84	4/59	1.45 (0.22, 9.63)	0.70	4/72	1.85 (0.18, 19.54)	0.61
5-<15	11/238	0.87 (0.39, 1.95)	0.74	14/67	0.96 (0.09, 9.73)	0.97	30/218	0.58 (0.22, 1.57)	0.29	18/139	1.89 (0.37, 9.85)	0.45	4/117	0.96 (0.09, 9.73)	0.97
≥15	23/54			1/1			10/55			2/20			1/19		
Transmission setting*															
Low transmission	57/168	0.64 (0.26, 1.54)	0.24	25/186	-	-	25/186	2.09 (0.75, 5.82)	0.16	12/78	0.67 (0.19, 2.38)	0.54	3/73	0.35 (0.06, 1.98)	0.24
Moderate to high transmission	111/231	Ref		19/114	Ref		20/130	Ref		12/140	Ref		6/135	Ref	

AOR, adjusted odds ratio; CI, confidence interval; PQ, primaquine;

Primaquine target dose; estimates adjusted for sex, age, hyperparasitaemia, log gametocytemia at baseline, schizontocidal treatment, baseline haemoglobin and transmission setting

*Primaquine target dose; estimates adjusted for sex, age, hyperparasitaemia, log gametocytemia at baseline, schizontocidal treatment and baseline haemoglobin

Table S6. Mixed effects logistic regression for probability of a patient infecting at least 1 mosquito and probability of a mosquito being infected in membrane experiments conducted on blood taken within 14 days from treatment in patients with gametocytaemia at baseline and at the time of sampling

	**Patient Infecting at least 1 Mosquito (N=531 feeds, n=251 patients, 3 Studies)**		**Mosquito gets infected (N = 30, 535 Mosquitoes, n = 531 Feeds, 251 Patients, 3 Studies)**	
	**AOR (95%CI)**	**P-value**	**AOR (95%CI)**	**P-value**
Effect of PQ dose over time, per day				
0.0625–0.125 mg/kg	0.53 (0.34, 0.83)	0.006	0.48 (0.33, 0.71)	<0.001
0.25 mg/kg	0.05 (0.01, 0.21)	<0.001	0.02 (0.01, 0.07)	<0.001
0.4-0.5 mg/kg	0.09 (0.02, 0.40)	0.002	0.12 (0.04, 0.37)	<0.001
Log10 gametocytemia at the time of sampling	6.09 (2.99, 12.37)	<0.001	7.05 (4.20, 11.83)	<0.001
ACT effect over time, per day				
AL	0.53 (0.35, 0.80)	0.003	0.54 (0.35, 0.80)	0.001
DP	0.94 (0.76, 1.19)	0.65	0.94 (0.76, 1.15)	0.53
Low transmission intensity	0.03 (0.002, 0.04)	0.007	0.06 (0.007, 0.57)	0.01

ACT, Artemisinin combination therapy; AL, artemether-lumefantrine; AOR, adjusted odds ratio; CI, confidence interval; DP, dihydroartemisinin-piperaquine; PQ, primaquine

Table S7. Baseline characteristics of patients included in haematology safety analysis.

	Age < 5 years (n=1169)				Age 5 -<15 years (n=2747)				Female age >= 15 years (n=494)				Male age >= 15 years (n=1362)			
	PQ		No PQ		PQ		No PQ		PQ		No PQ		PQ		No PQ	
**Parameter**	**N**	**n [%] or median [range]**	**N**	**n [%] or median [range]**	**N**	**n [%] or median [range]**	**N**	**n [%] or median [range]**	**N**	**n [%] or median [range]**	**N**	**n [%] or median [range]**	**N**	**n [%] or median [range]**	**N**	**n [%] or median [range]**
Sex: male	736	411 [55.8]	432	230 [53.2]	1875	1067 [56.9]	872	484 [55.5]	342	0 [0]	152	0 [0]	1112	1112 [100]	250	250 [100]
Age	737	3.0 [0.5 - 4.9]	432	3.0 [0.5 - 4.9]	1875	8.0 [5.0 - 14.8]	872	8.5 [5.0 - 14.0]	342	29.0 [15.0 - 79.0]	152	30.0 [15.0 - 84.0]	1112	27.4 [15.0 - 84.0]	250	25.0 [15.0 - 74.0]
Fever[1]	405	171 [42.2]	146	67 [45.9]	1348	402 [29.8]	511	155 [30.3]	286	155 [54.2]	136	75 [55.1]	976	495 [50.7]	228	133 [58.3]
Temperature (°C)	405	37.3 [36.0 - 40.7]	146	37.0 [36.0 - 40.4]	1348	36.9 [34.2 - 41.0]	511	36.8 [34.3 - 40.5]	286	37.8 [34.5 - 40.2]	136	37.8 [34.6 - 41.0]	976	37.6 [34.1 - 41.0]	228	38.0 [34.0 - 40.5]
Underweight[2]	695	80 [11.5]	421	54 [12.8]	47	13 [27.7]	20	1 [5.0]								
WAZ score	695	-0.7 [-4.1 - 7.8]	421	-0.7 [-3.8 - 14.1]	47	-1.0 [-3.6 - 2.0]	20	-0.4 [-2.4 - 1.2]								
Hb (g/dL)	737	10.3 [5.9 - 16.8]	432	10.2 [6.2 - 14.2]	1875	11.5 [6.0 - 17.7]	872	11.5 [7.0 - 16.4]	342	11.9 [7.1 - 17.8]	152	12.1 [8.7 - 16.7]	1112	13.4 [7.0 - 20.1]	1362	13.5 [7.0 - 20.1]
Anaemia																
No	737	448 [60.8]	432	250 [57.9]	1875	1592 [84.9]	872	746 [85.6]	342	311 [90.9]	152	140 [92.1]	1112	1074 [96.6]	250	245 [98.0]
Moderate-to-severe (<10g/dL)	737	279 [37.9]	432	171 [39.6]	1875	275 [14.7]	872	126 [14.4]	342	31 [9.1]	152	12 [7.9]	1112	38 [3.4]	250	5 [2.0]
Severe (<7g/dL)	737	10 [1.4]	432	11 [2.5]	1875	8 [0.4]	872	0 [0.0]	342	0 [0]	152	0 [0]	1112	0 [0]	250	0 [0]
G6PD status																
Normal	737	566 [76.8]	432	329 [76.2]	1875	1324 [70.6]	872	659 [75.6]	342	184 [53.8]	152	129 [84.9]	1112	328 [29.5]	250	221 [88.4]
Deficient	737	69 [9.4]	432	62 [14.4]	1875	106 [5.7]	872	96 [11.0]	342	7 [2.0]	152	4 [2.6]	1112	26 [2.3]	250	13 [5.2]
Intermediate	737	28 [3.8]	432	31 [7.2]	1875	29 [1.5]	872	32 [3.7]	342	0 [0.0]	152	0 [0.0]	1112	0 [0.0]	250	0 [0.0]
Unknown	737	74 [10.0]	432	10 [2.3]	1875	416 [22.2]	872	85 [9.7]	342	151 [44.2]	152	19 [12.5]	1112	758 [68.2]	250	16 [6.4]
Hyper-parasitaemia[3]	552	136 [24.6]	373	86 [23.1]	1382	200 [14.5]	693	74 [10.7]	262	15 [5.7]	119	5 [4.2]	966	101 [10.5]	215	6 [2.8}

Region																
Africa	737	679 [92.1]	432	430 [99.5]	1875	1495 [79.7]	872	789 [90.5]	342	163 [47.7]	152	113 [74.3]	1112	264 [23.7]	250	142 [56.8]
Asia	737	58 [7.9]	432	2 [0.5]	1875	380 [20.3]	872	83 [9.5]	342	179 [52.3]	152	39 [25.7]	1112	848 [76.3]	250	108 [43.2]
Transmission Intensity[4]																
Low	737	179 [24.3]	432	54 [12.5]	1875	973 [51.9]	872	337 [38.7]	342	341 [99.7]	152	151 [99.3]	1112	1086 [97.7]	250	238 [95.2]
Moderate	737	372 [50.5]	432	306 [70.8]	1875	619 [50.7]	872	442 [50.7]	342	1 [0.3]	152	1 [0.7]	1112	1 [0.1]	250	2 [0.8]
High	737	186 [25.2]	432	72 [16.7]	1875	283 [15.1]	872	93 [10.7]	342	0 [0]	152	0 [0]	1112	25 [2.3]	250	10 [4.0]
ACT																
AL	737	464 [63.0]	432	247 [57.2]	1875	865 [46.1]	872	369 [42.3]	342	109 [31.9]	152	40 [26.3]	1112	227 [20.4]	250	68 [27.2]
AL-AQ	737	29 [3.9]	432	0 [0.0]	1875	102 [5.4]	872	0 [0.0]	342	30 [8.8]	152	0 [0.0]	1112	125 [11.2]	250	0 [0.0]
ASSP	737	32 [4.3]	432	30 [6.9]	1875	82 [4.4]	872	80 [9.2]	342	50 [14.6]	152	51 [33.6]	1112	52 [4.7]	250	42 [16.8]
AS-MQ	737	0 [0.0]	432	0 [0.0]	1875	0 [0.0]	872	0 [0.0]	342	1 [0.3]	152	0 [0.0]	1112	72 [6.5]	250	0 [0.0]
DP	737	212 [28.8]	432	155 [35.9]	1875	796 [42.5]	872	423 [48.5]	342	124 [36.3]	152	61 [40.1]	1112	425 [38.2]	250	140 [56.0]
DP-MQ	737	0 [0.0]	432	0 [0.0]	1875	30 [1.6]	872	0 [0.0]	342	28 [8.2]	152	0 [0.0]	1112	211 [19.0]	250	0 [0.0]
Day of primaquine administration relative to start of ACT regimen																
Day 0	737	367 [49.8]			1875	664 [35.4]			342	62 [18.1]			1112	197 (17.7)		
Day 1	737	55 [7.5]			1875	251 [13.4]			342	122 [35.7]			1112	673 (60.5)		
Day 2	737	311 [42.2]			1875	864 [46.1]			342	86 [25.1]			1112	105 (9.4)		
Day 3	737	4 [0.5]			1875	96 [5.1]			342	72 [21.1]			1112	137 (12.3)		
Primaquine actual dose (mg/kg)	344	0.2 [0.1 - 0.8]			893	0.3 [0.0 - 1.9]			229	0.3 [0.2 - 1.4]			909	0.3 [0.1 - 1.4]		
Primaquine actual / target dose (mg/kg)	737	0.3 [0.1 - 0.8]			1875	0.3 [0.0 - 1.9]			342	0.3 [0.2 - 1.4]			1112	0.3 [0.1 - 1.4]		

[1]defined as temperature>37.5C or history of fever; [2] underweight is defined as waz score<-2; [3]defined as parasitaemia>100,000 parasites/µL; [4]Transmission Intensity areas defined based on estimates of P. falciparum prevalence rate (PfPR), assuming low transmission for study sites with a PfPR <0.15, moderate transmission if PfPR 0.15 to <0.40 and high transmission if PfPR ≥0.40;
AL =artemether-lumefantrine, ACT=artemisinin combination therapy, AL-AQ = artemether-lumefantrine-amodiaquine, ASSP= artesunate-sulfadoxine-pyrimethamine, AS-MQ= artesunate-mefloquine , DP=dihydroartemisinin-piperaquine. PQ- Primaquine,
N is total number of participants, n is number of events

Table S8. Patients with >25% fractional decrease in haemoglobin and/or anaemia at day 3 and day 7 in patients with haemoglobin $\geq$ 10 g/dl at baseline

	PQ				No PQ			
	**< 5 years**	**5-<15 years**	**Female >=15 years**	**Male >=15 years**	**< 5 years**	**5-<15 years**	**Female >=15 years**	**Male >=15 years**
**Day 3**	**N=414**	**N=1439**	**N=199**	**N=864**	**N=239**	**N=631**	**N=52**	**N=112**
Moderate-to-severe anaemia (Hb<10g/dL), % (n)	38.4 (159)	17.7 (254)	13.1 (26)	3.4 (29)	40.2 (96)	15.7 (99)	13.5 (7)	2.7 (3)
Severe Anaemia (Hb<7g/dL, % (n)	0.2 (1)	0.1 (1)	0 (0)	0 (0)	1.7 (4)	0 (0)	0 (0)	0 (0)
Fractional drop >25% & moderate-to-severe anaemia, % (n)	1.7 (7)	1.7 (25)	2.0 (4)	0.4 (3)	5.0 (12)	1.3 (8)	3.9 (2)	0.9 (1)
Fractional drop >25% & severe anaemia, % (n)	0.2 (1)	0.1 (1)	0 (0)	0 (0)	1.3 (3)	0 (0)	0 (0)	0 (0)
**Day 7**	**N=387**	**N=1330**	**N=173**	**N=367**	**N=237**	**N=708**	**N=115**	**N=197**
Moderate-to-severe anaemia (Hb<10g/dL), % (n)	26.6 (103)	12.6 (168)	9.3 (16)	3.5 (13)	30.8 (73)	9.8 (69)	6.1 (7)	2.0 (4)
Severe Anaemia (Hb<7g/dL, % (n)	0.3 (1)	0 (0)	0 (0)	0 (0)	0.8 (2)	0.1 (1)	0 (0)	0 (0)
Fractional drop >25% & moderate-to-severe anaemia, % (n)	2.6 (10)	1.9 (25)	2.3 (4)	0.8 (3)	4.2 (10)	1.4 (10)	2.6 (3)	1.0 (2)
Fractional drop >25% & severe anaemia, % (n)	0.3 (1)	0 (0)	0 (0)	0 (0)	0.8 (2)	0.1(1)	0 (0)	0 (0)

Table S9. Patients with >25% fractional decrease in haemoglobin or anaemia at day 3 and day 7 (including patients with baseline anaemia)

	PRIMAQUINE				No PRIMAQUINE			
	< 5 years	5-<15 years	Female >=15 years	Male >=15 years	< 5 years	5-<15 years	Female >=15 years	Male >=15 years
**Day 3**	**N=690**	**N=1697**	**N=220**	**N=897**	**N=416**	**N=742**	**N=60**	**N=113**
Moderate-to-severe Anaemia (Hb<10g/dL), % (n)	58.6 (404)	27.2 (461)	19.1 (42)	6.1 (55)	60.6 (252)	25.6 (190)	21.7 (13)	3.5 (4)
Severe Anaemia (Hb<7g/dL, % (n)	3.2 (22)	0.8 (14)	0.5 (1)	0 (0)	7.2 (30)	0.7 (5)	1.7 (1)	0 (0)
Fractional drop >25% & moderate-to-severe anaemia, % (n)	1.5 (10)	1.7 (28)	1.8 (4)	0.3 (3)	4.6 (19)	1.1 (8)	3.3 (2)	0.9 (1)
Fractional drop >25% & severe anaemia, % (n)	0.6 (4)	0.2 (4)	0 (0)	0 (0)	2.16 (9)	0 (0)	0 (0)	0 (0)
**Day 7**	**N=639**	**N=1533**	**N=188**	**N=377**	**N=409**	**N=829**	**N=123**	**N=202**
Moderate-to-severe Anaemia (Hb<10g/dL), % (n)	46.0 (294)	19.9 (310)	12.2 (23)	4.8 (18)	47.4 (194)	17.0 (141)	8.1 (10)	2.5 (5)
Severe Anaemia (Hb<7g/dL, % (n)	2.0 (13)	0.5 (8)	0.5 (1)	0.3 (1)	2.0 (8)	0.2 (2)	0 (0)	0 (0)
Fractional drop >25% & moderate-to-severe anaemia, % (n)	2.2 (14)	1.9 (29)	2.7 (5)	1.1 (4)	2.7 (11)	1.2 (10)	2.4 (3)	1.0 (2)
Fractional drop >25% & severe anaemia,% (n)	0.8 (5)	0.3 (4)	0.53 (1)	0.3 (1)	0.7 (3)	0.1 (1)	0 (0)	0 (0)

Figure S2. Proportion of patients at day 7 (A) with >25% fractional decrease in haemoglobin (B) Moderate-to-severe anaemia (Hb < 10g/dL) by G6PD status, and age/sex category

A. **Proportion of participants with >25% fractional decrease in haemoglobin**

G6PD Age (Years) G6PD Age (Years)

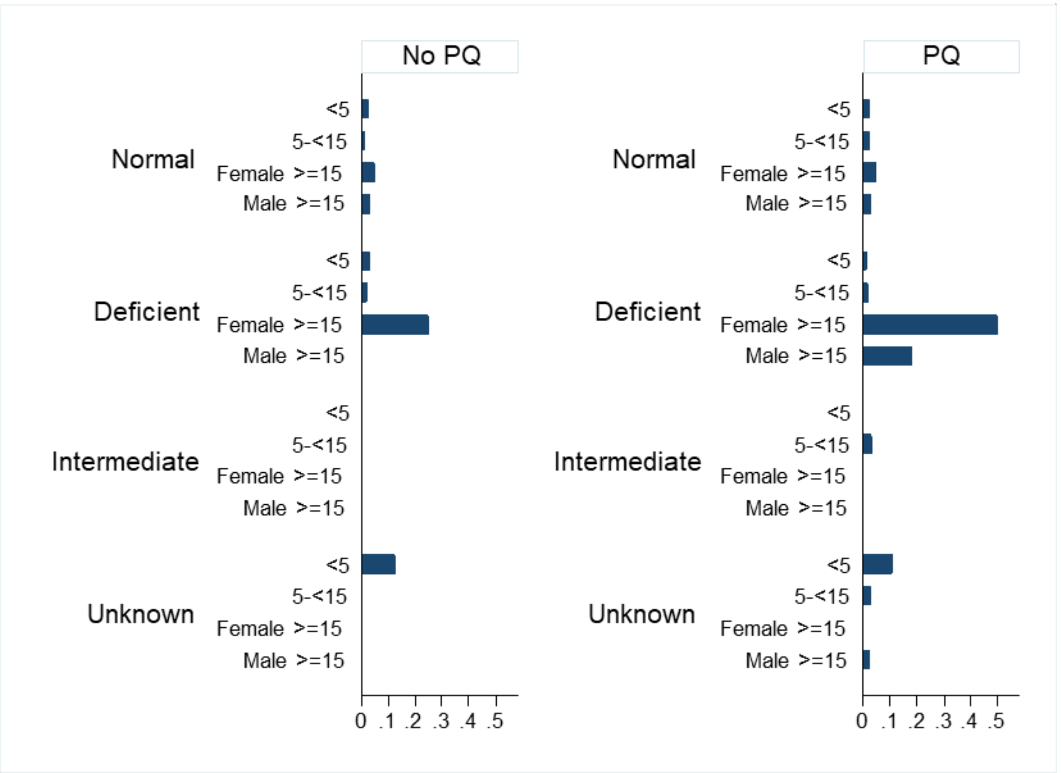

B. **Proportion of participants with moderate-to-severe anaemia (Hb < 10g/dL)**

G6PD Age (Years) G6PD Age (Years)

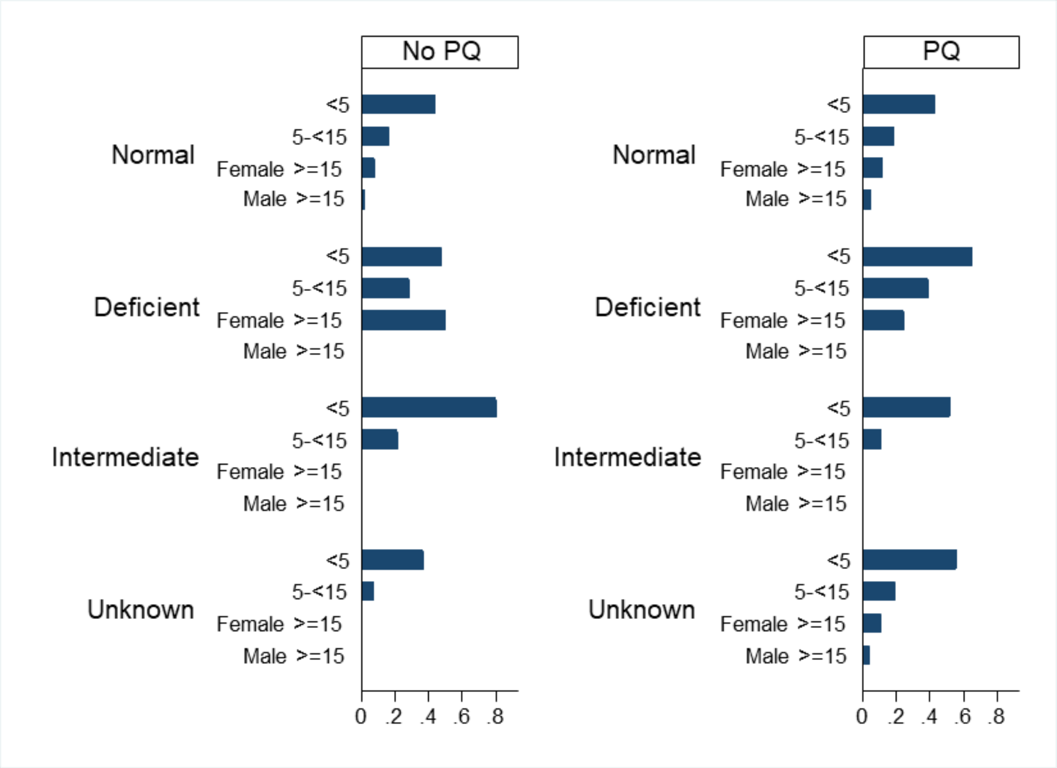

Table S10. Risk factors for change in haemoglobin concentration on day 7 after first dose of ACT administration

	**Absolute change in Hb (n=3960)**			**Moderate-to-severe Anaemia (Hb<10g/dL) (N=3960, n=869)**			**Severe Anaemia (Hb<7g/dL) (N=3960, n=26)****		
	**(g/dL)**	**95% CI**	**P-value**	**AOR**	**95% CI**	**P-value**	**AOR**	**95% CI**	**P-value**
**Age**[$]									
<5 years	-0.87	[-1.04,-0.71]	<0.001	2.88	[1.61,5.15]	<0.001			
5-<15 years	-0.62	[-0.76,-0.49]	<0.001	1.49	[0.87,2.59]	0.15			
Female >= 15 years	-0.63	[-0.79,-0.47]	<0.001	1.02	[0.53,1.98]	0.95			
Male >= 15 years	Ref			Ref		.			.
**Primaquine dose (0.1 mg/kg) by G6PD group**[#]									
Normal	-0.01	[-0.03,0.004]	0.14	1.04	[0.99,1.09]	0.09			
Deficient	-0.18	[-0.26,-0.09]	<0.001	1.33	[1.09,1.63]	0.006			
Intermediate	0.08	[-0.11,0.27]	0.40	0.68	[0.43,1.07]	0.10			
Unknown	-0.03	[-0.08,0.02]	0.30	1.14	[0.95,1.36]	0.14			
**Baseline Haemoglobin (g/dL)**	-0.45	[-0.47,-0.43]	<0.001	0.36	[0.33,0.39]	<0.001	0.29	[0.21,0.40]	<0.001
**Baseline Parasitic load (count/µL)**									
0*	Ref			Ref			Ref		
>0 and <100,000	0.03	[-0.13,0.19]	0.72	1.12	[0.69,1.80]	0.64	2.26	[0.47,10.84]	0.31
>100,000	-0.63	[-0.83,-0.43]	<0.001	3.49	[2.00,6.12]	<0.001	10.12	[1.82,56.29]	0.008
**Transmission Intensity**[$]									
Low	-0.06	[-0.22, 0.11]	0.50	2.01	[1.26,3.19]	0.003	4.26	[1.63,11.16]	0.003
Moderate-to-high	Ref			Ref					

AOR, adjusted odds ratio; CI, confidence interval,G6PD- Glucose-6-phosphate dehydrogenase, Hb-Haemoglobin
*Participants had gametocytes but no asexual forms at baseline
estimates and 95% CI are presented for the effect of 0.1 mg/Kg increase in primaquine in different G6PD status;
** No association was found between severe anaemia and age (P>0.5), G6PD status (P>0.2) and primaquine dose (P=0.20)
$ No significant difference was found on effect of primaquine dose (0.1 mg/kg) across age group and on different transmission setting (see Supplementary Table 11)

Table S11. The effect of primaquine dose on haemoglobin change across age group and transmission settings

	**Absolute change in Hb (n=3960)**			**Moderate-to-severe Anaemia (Hb<10g/dL) (N=3960, n=869)**			**Severe Anaemia (Hb< 7g/dL) (N=4320, n=33)**		
	**(g/dL)**	**95% CI**	**P-value**	**AOR**	**95% CI**	**P-value**	**AOR**	**95% CI**	**P-value**
**Primaquine dose (0.1 mg/kg) by age group***									
<5 years	-0.0005	[-0.03,0.03]	0.66	1.01	[0.95,1.09]	0.67	1.00	[0.79,1.27]	0.99
5-<15 years	-0.02	[-0.04,-0.0001]	0.01	1.04	[0.99,1.10]	0.12	1.23	[0.97,1.56]	0.08
Female >= 15 years	-0.03	[-0.07,0.01]	0.13	1.15	[0.98,1.36]	0.08	0.98	[0.41,2.33]	0.96
Male >= 15 years	0.01	[-0.02,0.05]	0.54	1.03	[0.85,1.24]	0.76	2.99	[0.31,28.67]	0.34
**Primaquine dose (0.1 mg/kg) by Transmission setting****									
Low	-0.01	[-0.02, 0.01]	0.58	1.04	[0.99,1.11]	0.15	1.03	[0.85,1.25]	0.74
Moderate-to-high	-0.02	[-0.05,-0.003]	0.08	1.03	[0.97,1.10]	0.32	1.07	[0.82,1.40]	0.63

* estimates adjusted for hyperparasitaemia at baseline, baseline haemoglobin and transmission setting

** estimates adjusted for age, hyperparasitaemia, and baseline haemoglobin

estimates adjusted for baseline haemoglobin

Figure S3. Mean with 95% Confidence interval haemoglobin 21 or 28 days after initiation of ACT

(In Glucose 6-Phosphate deficient (i) and normal (ii) participants that received primaquine (PQ) or no primaquine / placebo by age category (A) Age < 5 years; (B) Age 5-< 15 years ; (C) Age>= 15 years; (D) All ages)

i.G6PD deficient ii G6PD normal

A

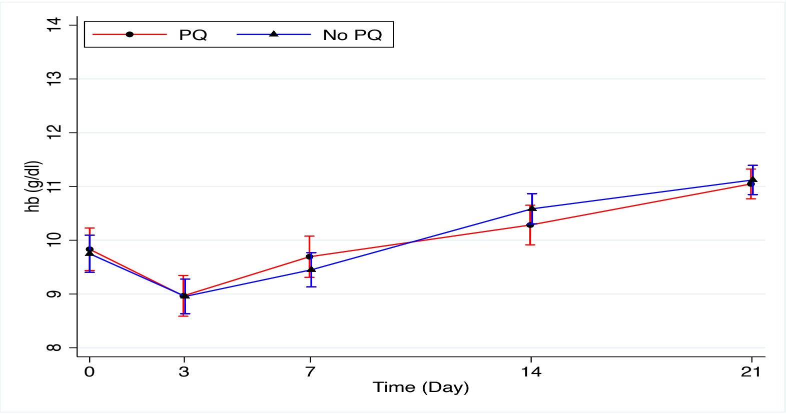

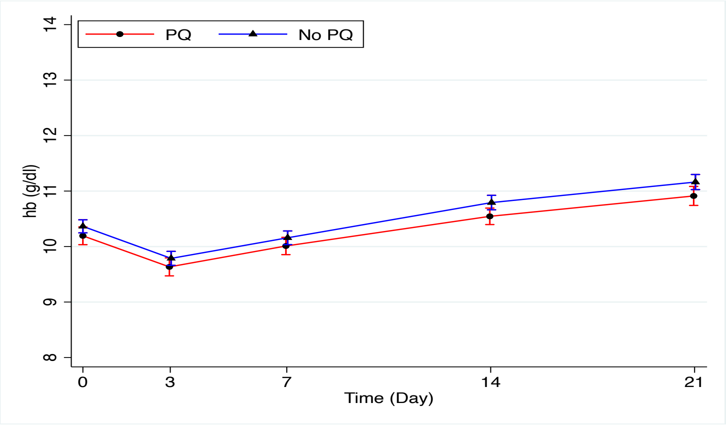

B

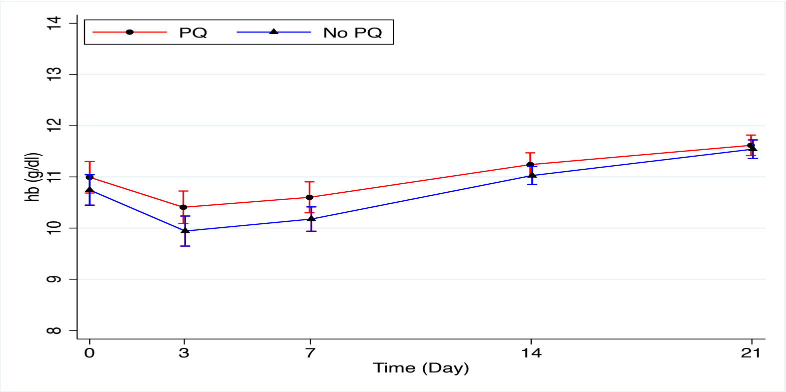

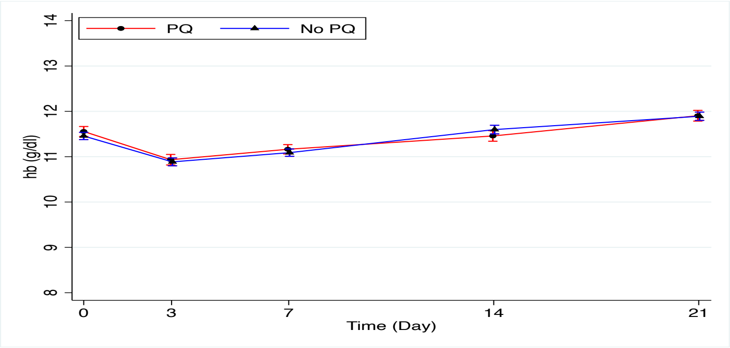

i.G6PD deficient ii.G6PD normal

C

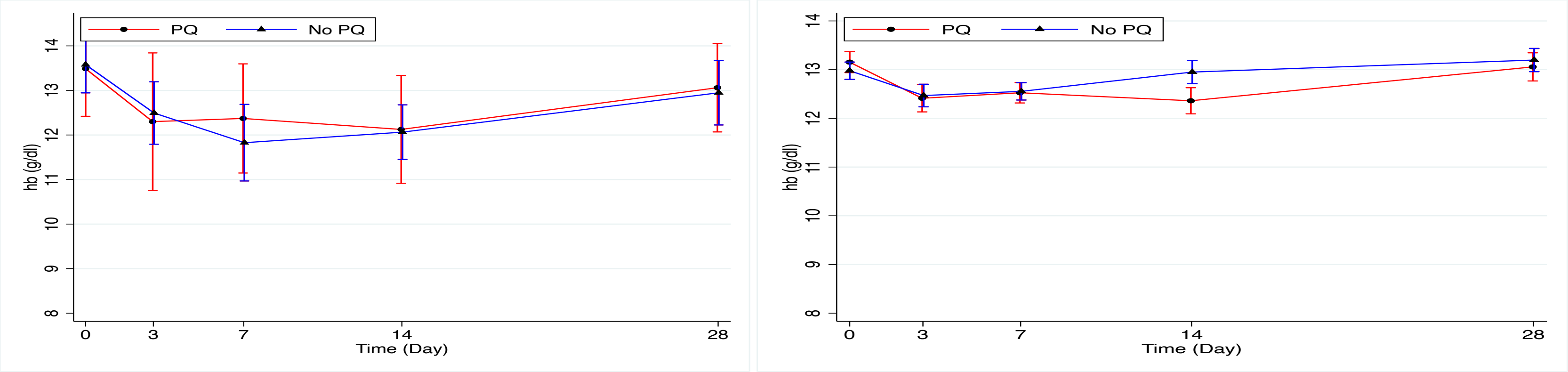
PQ
No PQ
hb (g/dl)
Time (Day)
0 3 7 14 28
8 9 10 11 12 13 14
PQ
No PQ
hb (g/dl)
Time (Day)
0 3 7 14 28
8 9 10 11 12 13 14

D.

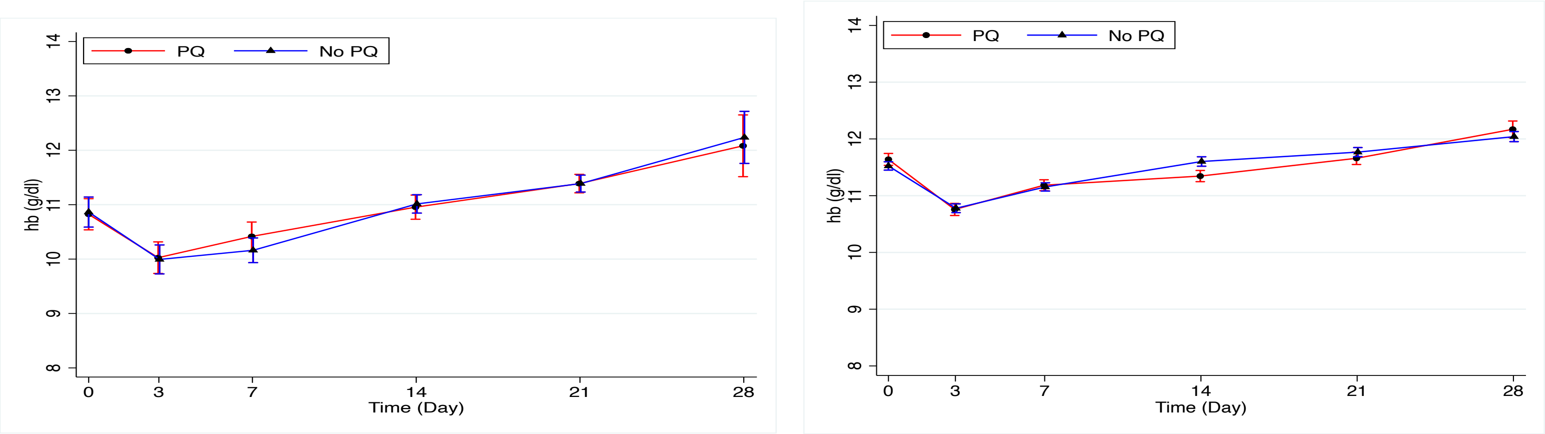
PQ
No PQ
hb (g/dl)
Time (Day)
0 3 7 14 21 28
8 9 10 11 12 13 14
PQ
No PQ
hb (g/dl)
Time (Day)
0 3 7 14 21 28
8 9 10 11 12 13 14

Table S12: Summary of adverse events by time since primaquine dosing and age categories in 9 controlled studies with a no-primaquine arm

	< 5 years						5-<15 years						>=15 years					
	PQ		No PQ				PQ		No PQ				PQ		No PQ			
	n	%	n	%	OR(95%CI)	P-value	n	%	n	%	OR(95%CI)	P-value	n	%	n	%	OR(95%CI)	P-value
**Day 3**	**6 studies with 300 patients**		**6 studies with 204 patients**				**9 studies with 896 patients**		**9 studies with 460 patients**				**6 studies with 261 patients**		**6 studies with 161 patients**			
Any AE	104	34.7	82	40.2	1.20 (0.79-1.82)	0.40	190	21.2	96	20.9	1.72 (1.25-2.37)	<0.001	47	18	27	16.8	1.47 (0.83-2.59)	0.18
Any SAE	10	3.4	6	2.9	1.33 (0.45-3.95)	0.61	1	0.1	1	0.2	0.51 (0.03-9.46)	0.65	1	0.4	1	0.6	0.93 (0.05-17.46)	0.96
Any AE > Grade 2*	40	16.8	33	17.5	1.27 (0.74-2.18)	0.38	47	8.9	52	15.4	0.76 (0.48-1.19)	0.23	11	7.2	8	6.5	1.30 (0.48-3.50)	0.61
Vomiting	33	11	26	12.7	1.33 (0.73-2.40)	0.35	25	2.8	28	6.1	0.72 (0.40-1.30)	0.28	3	1.1	3	1.9	0.92 (0.17-5.05)	0.93
Headache	2	0.7	3	1.5	1.11 (0.16-7.79)	0.92	19	2.1	6	1.3	1.28 (0.49-3.32)	0.61	12	4.6	6	3.7	1.24 (0.44-3.48)	0.69
Pyrexia	11	3.7	13	6.4	0.72 (0.30-1.74)	0.46	24	2.7	15	3.3	0.97 (0.48-1.94)	0.93	2	0.8	8	5	0.25 (0.05-1.32)	0.10
Abdominal pain	3	1	5	2.5	0.57 (0.12-2.65)	0.48	13	1.5	5	1.1	1.29 (0.44-3.79)	0.65	3	1.1	3	1.9	0.82 (0.15-4.45)	0.82
Any Gastrointestinal	39	13	34	16.7	1.20 (0.70-2.08)	0.51	38	4.2	33	7.2	0.83 (0.50-1.40)	0.49	10	3.8	6	3.7	1.49 (0.48-4.61)	0.49
**Day 7**	**6 studies with 300 patients**		**6 studies with 204 patients**				**9 studies with 896 patients**		**9 studies with 460 patients**				**6 studies with 261 patients**		**6 studies with 161 patients**			
Any AE	135	45	103	50.5	1.21 (0.80-1.83)	0.36	252	28.1	147	32	1.38 (1.03-1.85)	0.03	66	25.3	30	18.6	1.71 (1.02-2.88)	0.04
Any SAE	12	4.1	9	4.4	0.99 (0.39-2.53)	0.98	2	0.2	1	0.2	1.24 (0.11-14.42)	0.86	1	0.4	1	0.6	0.93 (0.05-17.46)	0.96
Any AE > Grade 2*	64	27.2	49	25.9	1.36 (0.85-2.18)	0.20	71	13.5	78	23.1	0.79 (0.53-1.17)	0.24	12	7.9	10	8.1	1.04 (0.41-2.60)	0.94
Vomiting	33	11	29	14.2	1.14 (0.64-2.04)	0.65	26	2.9	28	6.1	0.74 (0.41-1.33)	0.32	3	1.1	3	1.9	0.92 (0.17-5.05)	0.93
Headache	4	1.3	3	1.5	1.59 (0.30-8.38)	0.59	31	3.5	11	2.4	1.18 (0.57-2.42)	0.65	23	8.8	8	5	1.72 (0.73-4.07)	0.22
Pyrexia	17	5.7	13	6.4	1.08 (0.48-2.40)	0.86	33	3.7	21	4.6	0.87 (0.48-1.56)	0.63	3	1.1	8	5	0.35 (0.08-1.45)	0.15
Abdominal pain	3	1	5	2.5	0.57 (0.12-2.65)	0.48	17	1.9	8	1.7	0.96 (0.39-2.33)	0.93	10	3.8	3	1.9	2.05 (0.55-7.69)	0.29
Any Gastrointestinal	39	13	37	18.1	1.06 (0.62-1.82)	0.82	43	4.8	37	8	0.78 (0.48-1.28)	0.33	18	6.9	7	4.3	1.82 (0.69-4.79)	0.22

Day 28	4 studies with 229 patients		4 studies with171 patients				7 studies with 651 patients		7 studies with 351 patients				5 studies with 256 patients		5 studies with 157 patients			
Any AE	153	66.8	140	81.9	0.69 (0.38-1.27)	0.23	292	44.9	208	59.3	1.02 (0.72-1.44)	0.92	89	34.8	47	29.9	1.24 (0.80-1.91)	0.34
Any SAE	14	6.1	11	6.4	0.91 (0.38-2.17)	0.83	4	0.6	2	0.6	1.08 (0.20-5.92)	0.93	1	0.4	2	1.3	0.30 (0.03-3.38)	0.33

Table S13: Serious adverse events reported within 28 days of ACT with or without primaquine administration.

Study ID	Patient ID	Sex	Age, years	ACT	PQ	Day PQ started	G6PD status	Target PQ dose	Day AE started	AE preferred term	AE system organ class	Relatedness	Outcome
Nine RCTs with no-primaquine arm (n=2282)													
Day 0-3													
9	1991	-	4	DP	Yes	0	Normal	0.125	0	Vomiting	Gastrointestinal disorders	Possible	Recovered/resolved
13	38	Male	3	DP	Yes	0	Deficient	0.18	0	Anaemia (Hb 6.5 g/dl)*	Blood and lymphatic system disorders	Possible	Recovered/resolved
13	87	Male	4	AL	No	0	Normal	0	0	Anaemia (Hb 6.4 g/dl)*	Blood and lymphatic system disorders	Not related	Recovered/resolved
13	196	Female	2	DP	No	0	Normal	0	0	Tetanus	Bacterial infectious disorders	Not related	Recovered/resolved
13	419	Male	2	AL	No	0	Deficient	0	1	Anaemia (Hb 8.3 g/dl)*	Blood and lymphatic system disorders	Not related	Recovered/resolved
13	475	Male	4	DP	Yes	0	Normal	0.17	1	Anaemia (Hb 11.4 g/dl)*	Blood and lymphatic system disorders	Unlikely	Recovered/resolved
4	11	Male	3	AL	Yes	0	Normal	0.25	1	Haemolytic anaemia (Hb drop from 10.6 to 8.6 g/dl)	Blood and lymphatic system disorders	Not known	Recovered/resolved
4	170	Female	4	AL	Yes	0	Deficient	0.25	1	Anaemia (Hb drop from 8.2 to 6.8 g/dl)	Blood and lymphatic system disorders	Not known	Recovered/resolved
4	183	Male	1	AL	No	0	Deficient	0	1	Haemolytic anaemia (Hb drop from 8.1 to 6.6 g/dl)	Blood and lymphatic system disorders	Not known	Recovered/resolved
4	94	Female	30	AL	Yes	0	Normal	0.25	1	Haemolytic anaemia (Hb drop from 13.5 to 10.1g/dl)	Blood and lymphatic system disorders	Not known	Recovered/resolved
9	2054	Female	2	DP	Yes	0	Normal	0.25	1	Hb decreased (from 11.8 to 9.1g/dl)	Investigations	Possible	Recovered/resolved
9	2059	Male	2	DP	Yes	0	Deficient	0.25	1	Hb decreased (from 12.5 to 9.5 g/dl)	Investigations	Possible	Recovered/resolved
4	10	Female	23	AL	No	0	Deficient	0	2	Haemolytic anaemia (Hb drop from 8.7 to 8.6 g/dl)	Blood and lymphatic system disorders	Not known	Recovered/resolved
4	165	Female	2	AL	No	0	Normal	0	2	Anaemia (Hb drop from 8.8 to 6 g/dl)	Blood and lymphatic system disorders	Not known	Recovered/resolved
13	178	Female	7	AL	No	0	Normal	0	2	Anaemia (Hb drop from 9.4 to 4.9 g/dl)*	Blood and lymphatic system disorders	Unlikely	Recovered/resolved
13	380	Male	2	DP	Yes	0	Normal	0.21	2	Anaemia (Hb drop from 8 to 4.6 g/dl)*	Blood and lymphatic system disorders	Unlikely	Recovered/resolved

13	589	Female	3	AL	Yes	0	Normal	0.29	2	Anaemia (Hb drop from 6.8 to 4.8 g/dl)*	Blood and lymphatic system disorders	Not related	Recovered/resolved
2	5044	Female	4	DP	Yes	2	Normal	0.2	3	Injection site injury	General disorders and administration site conditions	Not related	Recovered/resolved
9	2051	Female	11	DP	Yes	0	Normal	0.4	3	Hb decreased (from 14.1 to 11 g/dl)	Investigations	Possible	Recovered/resolved
9	2056	Female	4	DP	No	0	Normal	0	3	Hb decreased (from 13.5 to 10.4 g/dl)	Investigations	Possible	Recovered/resolved
Day 4-7 (RCTs with no-primaquine arm)													
13	132	Female	9	AL	Yes	0	Deficient	0.17	4	Anaemia (Hb drop from 6.5 to 5.3 g/dl)*	Blood and lymphatic system disorders	Possible	Recovered/resolved
13	183	Male	5	AL	No	0	Deficient	0	4	Anaemia (Hb drop from 6.5 to 6.5 g/dl)*	Blood and lymphatic system disorders	Unlikely	Recovered/resolved
13	426	Male	4	DP	No	0	Normal	0	5	Anaemia (Hb drop from 9.8 to 7.5 g/dl)*	Blood and lymphatic system disorders	Unlikely	Recovered/resolved
9	2000	Male	3	DP	Yes	0	Normal	0.75	7	Hb decreased (from 10.4 to 7.4 g/dl)	Investigations	Possible	Recovered/resolved
9	2045	Male	5	DP	Yes	0	Normal	0.75	7	Hb decreased (from 15 to 10.5 g/dl)	Investigations	Unlikely	Recovered/resolved
13	131	Male	0.6	DP	No	0	Normal	0	7	Blood creatinine increased	Renal and urinary tract investigations and urinalyses	Not related	Recovered/resolved
Day 8-28 (RCTs with no-primaquine arm)													
18	SMKP095	Male	43	AL	No	3	Normal	0	9	Renal impairment	Renal and urinary disorders	Not related	Recovered/resolved
2	1184	Female	8	DP	Yes	2	Normal	0.2	10	Pneumonia	Infections and infestations	Unlikely	Recovered/resolved
2	1181	Male	11	DP	Yes	2	Normal	0.75	11	Pneumonia	Infections and infestations	Not related	Recovered/resolved
13	367	Male	10	AL	No	0	Normal	0	14	Diabetes mellitus	Glucose metabolism disorders (incl diabetes mellitus)	Not related	Not resolved
9	2062	Male	2	DP	Yes	0	Normal	0.75	15	Asthma	Respiratory, thoracic and mediastinal disorders	Not related	Recovered/resolved
13	40	Male	1	AL	No	0	Normal	0	20	Malaria	Protozoal infectious disorders	Not related	Recovered/resolved
9	2032	Male	4	DP	Yes	0	Normal	0.25	27	Pneumonia	Infections and infestations	Not related	Recovered/resolved
13	388	Female	3	AL	No	0	Normal	0	28	Aminotransferase increased	Hepatobiliary investigations	Not related	Recovered/resolved
13	388	Female	3	AL	No	0	Normal	0	28	Aminotransferase increased	Hepatobiliary investigations	Not related	Recovered/resolved
Four studies without a no-primaquine arm (n=1473)													
Day 0-3													
10	BANGLADESH001_2-053	Male	8	AL	Yes	1	Unknown	0.25	1	Haematocrit decreased	Investigations	Not described for PQ	recovered/resolved

10	BANGLADESH001_2-073	Male	6	AL	Yes	1	Unknown	0.25	0	Seizure	Nervous system disorders	Not described for PQ	recovered/resolved
10	CAMBODIA001_2-021	Male	27	As-MQ	Yes	1	Unknown	0.25	3	Aspartate aminotransferase increased	Investigations	Not described for PQ	recovered/resolved
10	CAMBODIA001_2-051	Male	33	DP-MQ	Yes	1	Unknown	0.25	0	Dyspnoea	Respiratory, thoracic and mediastinal disorders	Not described for PQ	recovered/resolved
10	CAMBODIA001_2-080	Male	30	As-MQ	Yes	1	Unknown	0.25	3	Haematocrit decreased	Investigations	Not described for PQ	recovered/resolved
10	CAMBODIA003_2-058	Male	39	DP	Yes	1	Unknown	0.25	1	Loss of consciousness	Nervous system disorders	Not described for PQ	recovered/resolved
10	INDIA004_2-050	Female	40	AL	Yes	1	Unknown	0.25	3	Blood creatinine increased	Investigations	Not described for PQ	recovered/resolved
10	INDIA005_2-006	Male	18	AL	Yes	1	Unknown	0.25	2	Electrocardiogram QT prolonged	Investigations	Not described for PQ	recovered/resolved
10	MYANMAR005_2-012	Male	52	DP-MQ	Yes	1	Unknown	0.25	2	Malaria	Infections and infestations	Not described for PQ	recovered/resolved
10	MYANMAR005_2-012	Male	52	DP-MQ	Yes	1	Unknown	0.25	1	Haematocrit decreased	Investigations	Not described for PQ	recovered/resolved
10	MYANMAR006_2-015	Male	14	DP-MQ	Yes	1	Unknown	0.25	3	Neutrophil count decreased	Investigations	Not described for PQ	recovered/resolved
10	MYANMAR006_2-017	Male	36	DP-MQ	Yes	1	Unknown	0.25	3	Neutrophil count decreased	Investigations	Not described for PQ	recovered/resolved
10	MYANMAR008_2-026	Male	17	DP-MQ	Yes	1	Unknown	0.25	1	Electrocardiogram QT prolonged	Investigations	Not described for PQ	recovered/resolved
10	THAILAND005_2-003	Male	37	DP	Yes	1	Unknown	0.25	2	Electrocardiogram QT prolonged	Investigations	Not described for PQ	recovered/resolved
10	THAILAND005_2-017	Male	55	DP	Yes	1	Unknown	0.25	2	Electrocardiogram QT prolonged	Investigations	Not described for PQ	recovered/resolved
10	VIETNAM001_2-020	Male	7	DP	Yes	1	Unknown	0.25	3	Electrocardiogram QT prolonged	Investigations	Not described for PQ	recovered/resolved
10	VIETNAM001_2-093	Female	45	DP	Yes	1	Unknown	0.25	0	Syncope	Nervous system disorders	Not described for PQ	recovered/resolved
10	VIETNAM001_2-094	Female	13	DP	Yes	1	Unknown	0.25	3	Unevaluable event	General disorders and administration site conditions	Not described for PQ	recovered/resolved
14	112	Male	28	AL	Yes	0	Normal	0.25	2	Malaria	Infections and infestations	not related	Fatal
14	29	Female	31	AL	Yes	0	Normal	0.25	1	Diarrhoea	gastrointestinal disorders	unlikely	recovered/resolved
14	29	Female	31	AL	Yes	0	Normal	0.25	1	Headache	nervous system disorders	unlikely	recovered/resolved
14	29	Female	31	AL	Yes	0	Normal	0.25	1	Vomiting	gastrointestinal disorders	unlikely	recovered/resolved
14	29	Female	31	AL	Yes	0	Normal	0.25	1	Dizziness	nervous system disorders	unlikely	recovered/resolved
14	79	Male	60	AL	Yes	0	Normal	0.25	0	Vomiting	gastrointestinal disorders	not related	Fatal
14	79	Male	60	AL	Yes	0	Normal	0.25	0	Diarrhoea	gastrointestinal disorders	not related	Fatal

14	79	Male	60	AL	Yes	0	Normal	0.25	0	Pyrexia	General disorders and administration site conditions	not related	Fatal
14	79	Male	60	AL	Yes	0	Normal	0.25	0	Chills		not related	Fatal
Day 8-28													
10	THAILAND005_2-004	Male	28	DP	Yes	0	Unknown	0.25	20	Plasmodium falciparum infection	Infections and infestations	Not described for PQ	recovered/resolved
10	THAILAND005_2-017	Male	55	DP	Yes	0	Unknown	0.25	28	Plasmodium falciparum infection	Infections and infestations	Not described for PQ	recovered/resolved

*Patients received blood transfusion in the first week of artemisinin-based combination therapy

One death detail in study 10 documented on published paper but not shared

Text S2. Supplementary results

Haemoglobinuria

Of seven studies (2981patients) that included haemoglobinuria data, three studies used direct questioning about dark urine, three used direct questioning and confirmation with urine dipstick, and one study used the Hillman test for monitoring haemoglobinuria. Only three studies reported any haemoglobinuria, which was detected in 23 (0.77% patients). It was more commonly reported in adults (2%, n=5) than young children (0.3%, n=15) and older children (0.8%, n=5) (p=0.02). Patients with G6PD deficiency (AOR 3.70, 95%CI: 1.40, 9.78) and hyperparasitaemia (> 100,000 count/ul) (AOR 3.54, 95%CI: 1.35, 9.34) had a higher risk of haemoglobinuria. Across all age groups, the risk of haemoglobinuria was similar in patients who received primaquine or no primaquine, and with each 0.1 mg/kg increase primaquine dose (Supplementary Figure S4 & Supplementary Table S14).

Figure S4. Forest plot for hemoglobinuria by age group

Study	Treatment Yes	Treatment No	Control Yes	Control No	RR with 95% CI	Weight (%)
**1. < 5 Years**						
The Gambia 2013	0	59	0	14	-1.39 [-5.27, 2.49]	5.53
Tanzania 2014	1	13	1	24	0.58 [-2.11, 3.27]	4.96
Burkina Faso 2013	0	55	1	26	-1.79 [-4.96, 1.38]	13.82
Kenya 2014a	0	23	0	7	-1.10 [-4.94, 2.74]	5.18
Uganda 2011	0	160	0	57	-1.02 [-4.93, 2.89]	5.08
Uganda 2023	0	251	0	263	0.05 [-3.87, 3.96]	3.38
Heterogeneity: I^2 = 0.00%, H^2 = 1.00					-0.66 [-1.93, 0.62]	
Test of $\theta_i = \theta_j$: Q(5) = 1.65, p = 0.90						
Test of θ = 0: z = -1.01, p = 0.31						
**2. 5-<15 years**						
The Gambia 2013	0	350	0	122	-1.05 [-4.96, 2.87]	5.12
Tanzania 2014	12	55	2	46	1.46 [0.01, 2.91]	16.11
Burkina Faso 2013	1	187	0	84	0.30 [-2.89, 3.49]	4.77
Kenya 2014a	0	13	0	3	-1.25 [-5.01, 2.51]	5.38
Uganda 2011	0	179	0	58	-1.12 [-5.02, 2.79]	5.21
Uganda 2023	0	318	0	303	-0.05 [-3.96, 3.87]	3.54
Mali 2013	0	40	0	6	-1.77 [-5.60, 2.07]	5.90
Heterogeneity: I^2 = 0.00%, H^2 = 1.00					0.61 [-0.31, 1.54]	
Test of $\theta_i = \theta_j$: Q(6) = 5.32, p = 0.50						
Test of θ = 0: z = 1.31, p = 0.19						
**3. >=15 Years**						
The Gambia 2013	0	108	0	38	-1.03 [-4.93, 2.88]	5.09
Tanzania 2014	3	26	1	36	1.34 [-0.87, 3.55]	6.07
Mali 2013	1	24	0	10	0.24 [-2.88, 3.36]	4.86
Heterogeneity: I^2 = 0.00%, H^2 = 1.00					0.67 [-0.83, 2.17]	
Test of $\theta_i = \theta_j$: Q(2) = 1.16, p = 0.56						
Test of θ = 0: z = 0.87, p = 0.38						
**Overall**					0.31 [-0.34, 0.95]	
Heterogeneity: I^2 = 0.00%, H^2 = 1.00						
Test of $\theta_i = \theta_j$: Q(15) = 9.93, p = 0.82						
Test of θ = 0: z = 0.93, p = 0.35						
Test of group differences: $Q_b(2)$ = 2.85, p = 0.24						

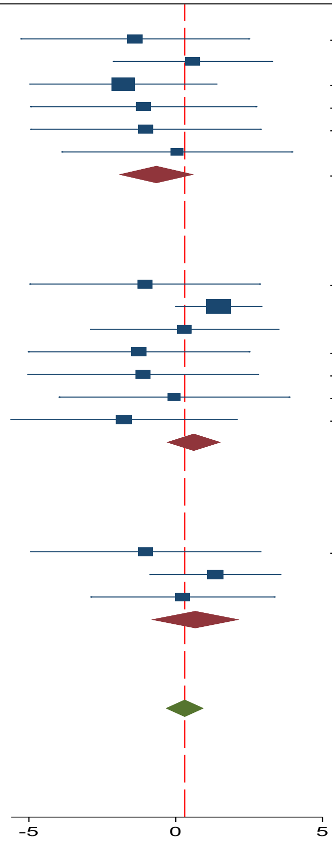

Fixed-effects Mantel–Haenszel model

Table S14. Factors associated with hemoglobinuria with logistic regression

	AOR	95% CI	P-value
**Primaquine dose (mg/kg)***	1.11	(0.15, 8.22)	0.92
**Age**			
<5 years	0.10	(0.02, 0.50)	0.005
5-<15 years	0.33	(0.10, 1.05)	0.06
>= 15 years	Ref		
**G6PD**			
Normal	Ref		
Deficient	3.70	(1.40, 9.78)	0.008
**Parasitic load (count//µL)**			
< 100,000	Ref		
> 100,000	3.54	(1.35, 9.34)	0.01

* Across all age groups the risk of haemoglobinuria was not associated with primaquine use, nor each 0.1 mg/kg increase in primaquine dose [AOR 0.04 (95% CI: 0.00, 164.76) for age < 5years, 1.41 (95% CI: 0.15, 13.08) for age 5-<15 years, and 0.35 (95% CI: 0.00, 28.67) for age ≥15years].

Table S15. Risk of bias assessment in randomised controlled studies

PMID	Bias from randomisation	Bias due to deviation from intervention	Bias from missing outcome			Bias in measurement of the outcome			Bias in selection of the reported results	Overall bias	Balanced age groups	Comparison of no PQ to PQ
			Efficacy	Haematology	AEs	Efficacy	Haematology	AEs				
29996844	Green	Green	Grey	Grey	Orange	Grey	Grey	Orange	Orange	Orange	Red	Red
27825738 & 25887344	Green	Green	Green	Green	Red	Green	Green	Red	Orange	Orange	Red	Green
27565897 & 27287612	Green	Green	Green	Green	Orange	Green	Green	Orange	Orange	Orange	Red	Green
28749756	Green	Red	Green	Grey	Grey	Green	Grey	Grey	Orange	Red	Red	Red
29548285	Green	Green	Grey	Green	Grey	Grey	Green	Grey	Orange	Orange	Red	Green
28289025 & 26952094	Green	Green	Green	Green	Red	Green	Green	Red	Orange	Orange	Red	Green
18074034	Green	Green	Green	Green	Grey	Green	Green	Grey	Orange	Orange	Red	Green
Unpublished	Green	Green	Green	Green	Orange	Green	Green	Orange	Orange	Orange	Red	Green
32171078 (final) & 31345710 (subset)	Green	Green	Grey	Green	Grey	Grey	Green	Grey	Orange	Orange	Red	Red
27197604 & 24239324 & 24913169	Green	Green	Green	Green	Grey	Green	Green	Grey	Orange	Orange	Red	Green
29324864	Green	Green	Green	Green	Orange	Green	Green	Orange	Orange	Orange	Red	Green
36462528	Green	Green	Grey	Green	Orange	Grey	Green	Orange	Orange	Orange	Red	Green
17925871	Green	Green	Green	Green	Grey	Green	Green	Grey	Orange	Orange	Red	Green
23175563	Green	Green	Grey	Green	Grey	Grey	Green	Grey	Orange	Orange	Red	Green
31234865	Green	Green	Green	Green	Red	Green	Green	Red	Orange	Orange	Red	Green
28931236	Green	Green	Green	Green	Grey	Green	Green	Grey	Orange	Orange	Red	Green
31964380	Green	Green	Green	Green	Red	Green	Green	Red	Orange	Orange	Red	Green
30871496 & 32179526	Green	Green	Green	Green	Grey	Green	Green	Grey	Orange	Orange	Red	Green
26906747	Green	Green	Green	Green	Orange	Green	Green	Orange	Orange	Orange	Red	Green

Green – low risk of bias; Red – high risk of bias; Orange – unclear risk of bias; Grey – not applicable; Assessed according to the Cochrane Risk of Bias 2 tool for randomised controlled trials; AEs – adverse events; PQ – primaquine

Table S16. Risk of bias assessment in single arm observational studies

PMID	Clear criteria for inclusion	Condition measured in reliable way	Valid methods for condition	Consecutive inclusion	Complete inclusion	Demographics reported	Clinical information reported	Outcomes reported	Site description	Analysis appropriate	Balanced age groups	Comparison of no PQ to PQ
Unpublished												
27450652												
27036739												
27128675												

Green – yes (low risk of bias); Red – no (higher risk of bias); Orange – unclear; Grey – not applicable; Assessed according to the Joanna Briggs Institute Case Series tool for single arm studies; the appropriateness of analysis was considered appropriate for all studies given that the individual patient data were re-analysed as part of these meta-analyses; PQ – primaquine.

Table S17. Eligible studies not included

First Author-Year	Design	Country	Region	Follow up (days)	Treatment arms	patients enrolled	Target PQ dose	Female (%)	Mean Age (SD)	Median Age (range)	Reasons for exclusion	Eligibility			
												Efficacy	Membrane feeding	Haem	Adverse events
K. Congpuong-2010[5]	Cohort	Thailand	Asia-Pacific	42	ASMQ	51	0.6	17.8		30 (6,80)	No response	No	No	No	Yes
K. Congpuong-2010[6]	Cohort	Thailand	Asia-Pacific	42	ASMQ	240	0.6	18.3		27 (4,69)	No response	No	No	No	Yes
Leang R-2019[7]	Cohort	Cambodia	Asia-Pacific	42	ASPYR	121	Not described	17.4		15 (7, 64)	No response	No	No	No	Yes
Rahman R-2016[8]	Cohort	Guyana	South - Americas	28	AS	50	0.5	2.0	30.6 (12–58)		No response	No	No	No	Yes
Mishra N-2014[9]	Cohort	India	Asia-Pacific	42	ASSP	175	0.75	42.9	Not stated		No response	No	No	No	Yes
Mendes Jorge M-2019[10]	RCT	Burkina Faso	Africa	28	ASAQ	100	Based on age	49.0		42 (10, 59)	No response	No	No	Yes	Yes
Hamaluba M-2021[11]	RCT	Kenya	Africa	42	Artp, ArtpMQ, AL	217	0.25	48.0		7.1 (-)	IPD not shared	No	No	No	Yes
Stone W-2022[12]	RCT	Mali	Africa	28	ASPYR, DP	100	0.25	52.0	Not stated		Ongoing in original search	No	Yes	Yes	Yes

AL – artemether-lumefantrine; Artp – arterolane piperaquine; ArtpMQ – arterolane piperaquine mefloquine; AS – artesunate; ASMQ – artesunate mefloquine; ASPYR – artesunate pyronaridine; ASSP– artesunate sulfadoxine pyrimethamine ;DP – dihydroartemisinin-piperaquine; IPD– individual participant data; PQ– primaquine; SD – standard deviation; RCT– randomized control trial;

References

1. Gething PW, Patil AP, Smith DL, Guerra CA, Elyazar IR, Johnston GL, et al. A new world malaria map: Plasmodium falciparum endemicity in 2010. Malar J. 2011 Dec 20;10:378. doi: 10.1186/1475-2875-10-378. PMID: 22185615; PMCID: PMC3274487.
2. IDDO/WWARN Policy on Data Use, Publication and Credit. October 2024 https://www.iddo.org/document/publication-policy
3. Higgins JPT, Savović J, Page MJ, Elbers RG, JAC S. Chapter 8: Assessing risk of bias in a randomized trial. In: Higgins JPT, Thomas J, Chandler J, et al., eds. Cochrane Handbook for Systematic Reviews of Interventions version 63 (updated February 2022): Cochrane; 2022. [Internet]. [cited 2024 Sep 12]. Available from: https://training.cochrane.org/handbook/current/chapter-08
4. Munn Z, Moola S, Lisy K, Riitano D, Tufanaru C. Methodological guidance for systematic reviews of observational epidemiological studies reporting prevalence and cumulative incidence data. Int J Evid Based Healthc. 2015 Sep;13(3):147–53.
5. Congpuong K, Saipomsud W, Chompoonuch C, Niemhom P, Vinayak S, Satimai W. Efficacy of a 3-day artesunate-mefloquine combination in the treatment of uncomplicated falciparum malaria in Kanchanaburi province of Thailand. Asian Biomed. 2010 Apr 1;4(2):289–95.
6 Congpuong K, Bualombai P, Banmairuroi V, Na-Bangchang K. Compliance with a three-day course of artesunate-mefloquine combination and baseline anti-malarial treatment in an area of Thailand with highly multidrug resistant falciparum malaria. Malar J. 2010 Feb 4;9:43.
7. Leang R, Mairet-Khedim M, Chea H, Huy R, Khim N, Mey Bouth D, et al. Efficacy and Safety of Pyronaridine-Artesunate plus Single-Dose Primaquine for Treatment of Uncomplicated Plasmodium falciparum Malaria in Eastern Cambodia. Antimicrob Agents Chemother. 2019 Mar;63(3):e02242-18.
8. Rahman R, Martin MJS, Persaud S, Ceron N, Kellman D, Musset L, et al. Continued Sensitivity of Plasmodium falciparum to Artemisinin in Guyana, With Absence of Kelch Propeller Domain Mutant Alleles. Open Forum Infect Dis. 2016 Sep;3(3):ofw185.
9. Mishra N, Kaitholia K, Srivastava B, Shah NK, Narayan JP, Dev V, et al. Declining efficacy of artesunate plus sulphadoxine-pyrimethamine in northeastern India. Malar J. 2014 Jul 22;13:284.
10. Mendes Jorge M, Ouermi L, Meissner P, Compaoré G, Coulibaly B, Nebie E, et al. Safety and efficacy of artesunate-amodiaquine combined with either methylene blue or primaquine in children with falciparum malaria in Burkina Faso: A randomized controlled trial. PloS One. 2019;14(10):e0222993.
11. Hamaluba M, van der Pluijm RW, Weya J, Njuguna P, Ngama M, Kalume P, et al. Arterolane-piperaquine-mefloquine versus arterolane-piperaquine and artemether-lumefantrine in the treatment of uncomplicated Plasmodium falciparum malaria in Kenyan children: a single-centre, open-label, randomised, non-inferiority trial. Lancet Infect Dis. 2021 Oct;21(10):1395–406.
12. Stone W, Mahamar A, Sanogo K, Sinaba Y, Niambele SM, Sacko A, et al. Pyronaridine-artesunate or dihydroartemisinin-piperaquine combined with single low-dose primaquine to prevent Plasmodium falciparum malaria transmission in Ouélessébougou, Mali: a four-arm, single-blind, phase 2/3, randomised trial. Lancet Microbe. 2022 Jan;3(1):e41–51.
